# Green Synthesis and Application of Platinum-Based Catalysts for Fuel Cells

**DOI:** 10.3390/molecules31101562

**Published:** 2026-05-08

**Authors:** Jiaxing Zhang, Hongbiao Ling, Weixu Wang, Chao Wang, Junjun Zhao, Xinyue Qiu, Zhen Lu, Haidong Zhao

**Affiliations:** 1 School of Chemistry and Chemical Engineering, Shanxi Datong University, Datong 037009, China; 13509735644@163.com (J.Z.); linghongbiao2024@163.com (H.L.); wang_wx10@163.com (W.W.); 18434368919@163.com (C.W.); zhao0000006@126.com (J.Z.); qxy202504@163.com (X.Q.); luzhen0313@aliyun.com (Z.L.); 2 Shanxi Province Union Laboratory of Clean Energy Materials, Shanxi Datong University, Datong 037009, China

**Keywords:** fuel cells, platinum-based catalysts, green synthesis, structure–activity relationship

## Abstract

Fuel cells are regarded as highly promising energy devices due to their clean and efficient energy conversion characteristics. However, their core material, platinum-based catalysts face challenges such as high cost, resource scarcity, and the high energy consumption and pollution associated with traditional synthesis methods, which contradict the green development principles of fuel cell technology. The rise of green chemistry provides a new research direction for developing environmentally friendly and cost-effective catalyst preparation routes. This review systematically summarizes recent research progress in the green synthesis of platinum-based catalysts for fuel cells, focusing on four core strategies: green solvent systems, biological reduction systems, renewable resource templates, and green energy-saving methods. It provides a detailed analysis of the principles of each method and their regulatory mechanisms on the microstructure. More importantly, this review elucidates the effects of size, morphology, and surface state on catalytic performance and establishes a structure–activity relationship linking green synthesis methods, microstructure, and catalytic performance and further discusses the regulatory mechanisms of catalyst structure, operating temperature, and electrolyte environment on electrochemical kinetic behavior. Furthermore, this article critically evaluates the advantages, limitations, and industrialization challenges of various green technologies. This review provides an important reference for the preparation and industrial application of high-performance, low-platinum, and environmentally friendly fuel cell catalysts.

## 1. Introduction

The overreliance on fossil fuels has led to energy depletion and global environmental degradation. Therefore, developing efficient and low-carbon energy conversion technologies has become a central issue for sustainable development [1,2]. Against this backdrop, fuel cell technology is widely recognized as a highly promising sustainable energy solution. This technology directly converts the chemical energy of fuel into electricity with high efficiency and features clean characteristics, including low or even zero emissions, showing great potential in areas such as transportation and stationary power generation [3,4,5].

**Figure 1:**
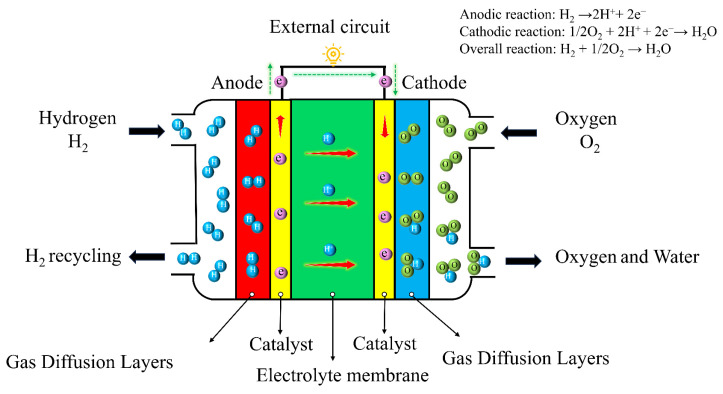
A schematic diagram of a fuel cell.

The primarily structure of a fuel cell consists of three components: electrodes, electrolyte membrane, and external circuit. As shown in Figure 1, the anode is responsible for the electrochemical oxidation of fuel, the cathode provides a site for the reduction reaction of oxidants [6,7]. The core electrochemical reaction kinetics in fuel cells are slow and highly dependent on efficient electrocatalysts [8,9,10,11]. However, platinum has an extremely low global reserve and a high cost. Moreover, conventional Pt/C catalysts are prone to issues such as nanoparticle agglomeration, Ostwald ripening, carbon support corrosion, and platinum dissolution. These problems lead to activity degradation, which significantly hinders the cost reduction and large-scale commercialization of fuel cells [12]. The preparation of traditional catalysts primarily relies on methods such as chemical reduction, impregnation, polyol synthesis, sol-gel synthesis, and pulsed laser ablation [13,14,15,16,17]. These methods typically require the use of toxic or flammable organic solvents, strong reducing agents, and complex surfactants or capping agents. Their synthesis processes are often energy-intensive and generate harmful byproducts. This results in a significant environmental burden and subsequent disposal costs, which contradict the green principles of fuel cell technology itself [18,19,20].

To address this challenge, the twelve principles of green chemistry provide a systematic framework for fundamentally reforming material preparation processes [21,22]. Applying these principles to the synthesis of platinum-based catalysts aims not only to reduce environmental burden but also to achieve controlled regulation of nanostructures through clean and precise synthetic strategies. This approach reduces platinum loading while enhancing mass activity (MA) and long-term stability, thereby advancing fuel cells toward high performance, low cost, and large-scale industrial development [23,24,25,26].

This review comprehensively summarizes recent advances in the green synthesis techniques of platinum-based catalysts. It first outlines four core green strategies: green solvent systems, biosynthesis systems, renewable resource templates, and energy-efficient methods, with an emphasis on analyzing their principles and structure-regulation mechanisms. Second, it explores the regulatory effects of green synthesis on catalyst size, morphology, and surface state, establishing a structure–performance relationship, and further discusses the regulatory mechanisms of catalyst structure, operating temperature, and electrolyte environment on electrochemical kinetic behavior. Finally, it identifies key scientific issues and industrialization challenges in the field, and outlines future development directions. This review serves as an important reference for the rational design and industrial application of high-performance, low-cost, and sustainable platinum-based catalysts.

## 2. Green Synthesis, Performance Regulation and Rational Design of Platinum-Based Catalysts for Fuel Cells

To overcome the problems of high cost and heavy environmental burden associated with traditional preparation methods, it is imperative to develop green and sustainable synthesis strategies. This section systematically reviews the current mainstream green preparation methods, categorizing them into four major types based on their core principles: green solvent systems, biological reduction systems, renewable resource templates, and green energy-saving methods. These methods not only aim to minimize environmental burden and reduce production costs, but also enable precise control over the size, morphology, dispersion, and surface state of nanoparticles, providing new ideas for the preparation of high-performance catalysts with low platinum loading. Furthermore, this section compares the performance of green-synthesized catalysts with that of conventional catalysts, discusses strategies for optimizing platinum loading and employing alternative metals such as palladium, demonstrates how DFT calculations elucidate catalytic mechanisms, and outlines the development directions for next-generation electrocatalysts.

### 2.1. Green Solvent Systems

Eliminating toxic and volatile organic solvents is the primary principle of green synthesis. Green solvent systems aim to identify safe, inexpensive, and renewable reaction media, while simultaneously achieving precise control over catalyst nanostructures. Their evolution has expanded from the most common solvent, water, to supercritical fluids, inorganic molten salts, and deep eutectic solvents, each with unique physicochemical properties. This progression reflects the pursuit of higher efficiency, enhanced structure-regulation capability, and improved industrial feasibility.

#### 2.1.1. Green Aqueous Reduction Method

Using water as the primary reaction medium is the most direct and economical green strategy. The core challenge lies in achieving controlled reduction and crystal growth of noble metal precursors under mild conditions while preventing particle agglomeration. In recent years, researchers have designed ingenious green reduction-oriented systems and achieved significant progress.

**Figure 2:**
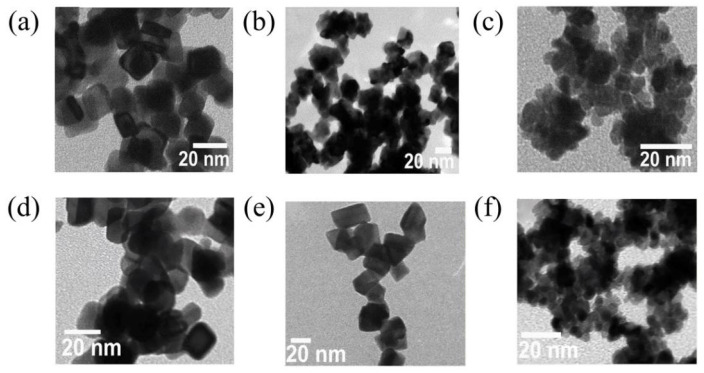
Transmission Electron Microscopy (TEM) images of Pt__0.21_, Pt__3.11_ and Pt__5.20_ particles synthesized at acetic acid concentrations of 0.21 M (**a**), 3.11 M (**b**) and 5.20 M (**c**), as well as Pt_3_Pd1__0.21_, Pt_3_Pd_1_3.11_ and Pt_3_Pd_1_5.20_ particles synthesized at 0.21 M (**d**), 3.11 M (**e**) and 5.20 M (**f**) [27]. Copyright 2016, The Royal Society of Chemistry.

**Figure 3:**
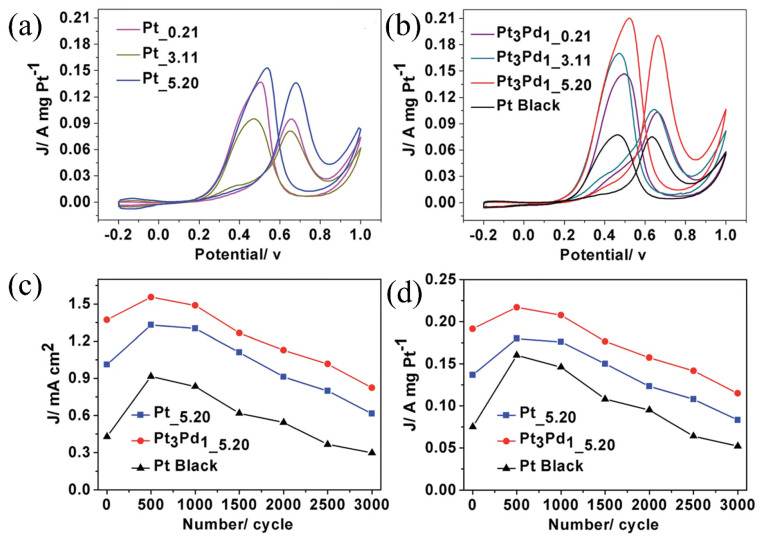
(**a**,**b**) mass-normalized initial Cyclic Voltammetry (CV) of Pt__0.21_, Pt__3.11_ and Pt__5.20_, Pt_3_Pd_1_0.21_, Pt_3_Pd_1_3.11_ and Pt_3_Pd_1_5.20_ NPs and the commercial Pt black in 0.5 M HClO_4_ and 1 M CH_3_OH solution; (**c**) typical ECSA-normalized CVs and (**d**) mass-normalized of Pt__5.20_, Pt_3_Pd_1_5.20_ and the commercial Pt black in 0.5 M HClO_4_ and 1 M CH_3_OH solution at different cycle number [27]. Copyright 2016, The Royal Society of Chemistry.

Zhang et al. [27] employed acetic acid as the sole green medium, which simultaneously served as the solvent, structure-directing agent, and precursor of an in situ reducing agent, without the addition of any surfactants or external reducing agents. The reaction was carried out under hydrothermal conditions at 180 °C for 2 h. Based on the detection of characteristic peaks of acetaldehyde in the Fourier Transform Infrared (FTIR) spectrum of the supernatant after the reaction, the authors speculated that acetic acid thermally decomposed to generate H_2_ in situ (the actual reducing agent), which subsequently reduced Pt^4+^ and Pd^2+^ to Pt and PtPd alloy. By adjusting the acetic acid concentration, “naked” Pt and PtPd alloy nanoparticles with clean surfaces and controllable morphologies were successfully synthesized, including well-defined polyhedrons, irregular polyhedron-constructed chain networks, and nanoflower-assembled chain networks (Figure 2). FTIR measurements after washing showed no significant organic residues on the catalyst surface, confirming its clean surface characteristics. As shown in Figure 3a,b, the Pt__5.20_ and Pt_3_Pd_1_5.20_ catalysts, which possessed a nanoflower-assembled chain network structure (5.20 indicated the molar concentration of acetic acid used in the catalyst preparation), exhibited mass activities for methanol oxidation that were 1.83 and 2.52 times higher than that of commercial Pt black, respectively. After 3000 accelerated cycles, Pt_3_Pd_1_5.20_ showed significantly enhanced stability compared to the commercial catalyst, with a much lower degree of activity decay (Figure 3c,d). The excellent performance was attributed to two factors: on the one hand, the weak adsorption and easy removal of acetic acid provided the catalyst with a clean surface, effectively avoiding the blocking of active sites by organic additives; on the other hand, the three-dimensional chain network structure constructed by nanoflowers exposed a high density of surface active sites (steps, edges, and kinks) and optimized interfacial mass transfer, thereby enhancing the catalytic reaction kinetics.

Xu et al. [28] developed and improved an in situ overgrowth strategy, using ethanol as a green promoter to enhance the reduction capacity of Graphene Oxide (GO), thereby forming ultra-clean, uniformly dispersed petal-shaped PtPd alloy nanostructures on the GO surface. This catalyst exhibited excellent low-temperature adaptability in alkaline methanol oxidation reactions. It retained approximately 70% of its activity at 5 °C, which was significantly higher than the 57% retained by commercial Pt/C. Benefiting from the electronic synergy and bifunctional mechanism of the PtPd alloy, its MA reached 924 mA/mg_Pt_, and it maintained 56.7% of its initial activity after 10,000 s of durability testing, making it suitable for fuel cell applications in extremely cold environments. Hanifah et al. [29] synthesized a ternary RGO/Pt-Pd alloy/CeO_2_ nanocomposite system via a one-step hydrothermal method. The prepared Pt-Pd alloy nanoparticles were spherical, exhibited excellent dispersibility, and were uniformly distributed on the surface and edge folds of the RGO. The electrochemical Active Surface Area (ECSA) values of the RGO/Pt-Pd alloy/CeO_2_, RGO/Pt/CeO_2_ and RGO/Pd/CeO_2_ catalysts were 0.90 cm^2^, 0.56 cm^2^, and 0.29 cm^2^, respectively. Combined with the bifunctional effect of CeO_2_ that promotes the oxidation of poisoning intermediates, the catalyst achieved a maximum forward peak current density of 69.82 mA/cm^2^ for methanol oxidation, which was approximately 3 times that of RGO/Pt/CeO_2_ and 50 times that of RGO/Pd/CeO_2_. Its onset potential was as low as 0.37 V, and the ratio of forward peak current to backward peak current (*I_f_*/*I_b_*) reached up to 2.29, demonstrating outstanding anti-poisoning capability. Meanwhile, after a 3000 s durability test, the catalyst still retained a residual current density of 0.37 mA·cm^−2^ and exhibited excellent electron transport efficiency.

However, it still has notable limitations: poor water solubility of some platinum precursors leads to low reaction rates, insufficient precision in controlling crystal growth, agglomeration tends to occur under high current density, and the ability to regulate the surface electronic structure of the catalyst is limited. Therefore, further performance enhancement typically requires combining with alloying or support modification.

#### 2.1.2. Supercritical Fluids

Supercritical fluids, particularly supercritical carbon dioxide (scCO_2_), possess the characteristics of low viscosity and high diffusivity of gases as well as high solubility of liquids. Additionally, they exhibit near-zero surface tension, allowing them to penetrate porous supports such as graphene and carbon black without damaging their fine structures [30,31]. In addition, scCO_2_ is non-toxic, non-flammable, and recyclable. After the reaction, it can be separated simply by depressurization, simplifying the process to three steps: dissolution, adsorption, and reduction. This approach avoids the emission of organic solvents and the high-energy drying and calcination steps at the source, aligning well with the principles of green chemistry and sustainable development [32,33].

Lin et al. [34] used supercritical CO_2_ as a green reaction medium to reduce the Pt(acac)_2_ precursor with H_2_, and successfully deposited Pt nanoparticles with a uniform size (5–10 nm) and a loading of about 25 wt% on the surface of carbon nanotubes (CNT). This method avoided the tedious steps of traditional wet-chemical processes and demonstrated the potential of a clean and efficient approach for catalyst preparation. In the oxygen reduction reaction (ORR), the as-prepared catalyst exhibited a Tafel slope of −21 mV/decade, and its exchange current density was one order of magnitude higher than that of commercial Pt/C. The *I_f_*/*I_b_* ratio for the methanol oxidation reaction (MOR) reached 1.3–1.6. The Pt-CNT catalyst showed excellent catalytic activity and poisoning resistance. Building on this work, Daş et al. [35,36] conducted a series of systematic studies. They [35] first compared the performance differences of Pt catalysts supported on graphene nanoplatelets (GNPs) prepared by the scCO_2_ deposition method versus the microwave irradiation method. The results showed that the Pt/G2 catalyst prepared by the scCO_2_ method had a particle size of only 1.5–1.6 nm with highly uniform dispersion, the ECSA of 87.2 m^2^/g, and an activity loss of 41.1% after 24 h of 1.2 V constant-voltage accelerated carbon corrosion aging. In contrast, the Pt/G1 catalyst prepared by the microwave method had a larger particle size of 3.1 to 3.4 nm, a much lower ECSA of 24.3 m^2^/g, an activity loss of 55.7% after the same aging test, and a Tafel slope loss of 50 mV. Therefore, the carbon corrosion resistance and Tafel slope stability of Pt/G2 were significantly better than those of Pt/G1. Subsequently, Daş et al. [36] extended the scCO_2_ technique to platinum-based alloy systems. They employed a simultaneous deposition strategy to in situ co-reduce Pt precursors with Ni, Fe, and Cu precursors, successfully preparing ultra-small alloy particles ranging from 1.6 to 2.1 nm. The PtNi/GNPs catalyst exhibited the ECSA of 132 m^2^/g_Pt_ and a half-wave potential of 0.79 V for the ORR. During the accelerated durability test (ADT), its ECSA loss rate was only 7–9%, demonstrating a durability that was twice that of PtFe/GNPs and four times that of PtCu/GNPs. In proton exchange membrane fuel cell (PEMFC) tests, this catalyst delivered a power density of 0.502 W/cm^2^ at 0.6 V. This work filled the research gap regarding the simultaneous deposition of Pt-based alloys using scCO_2_ in the PEMFC field.

Despite the significant advantages of scCO_2_ technology in particle dispersion, microstructure control, and CO_2_ utilization, its industrialization still faces multiple challenges. First, the solubility of platinum-based precursors in non-polar scCO_2_ is low, often requiring fluorinated ligands or co-solvents, which can easily introduce impurities. Second, under high-pressure and high-temperature conditions, achieving a precise balance between particle size and metal loading is difficult. Meanwhile, the high capital cost of specialized high-pressure equipment remains an issue, and challenges related to pressure stability and uniform mass transport in large-scale production have yet to be resolved.

#### 2.1.3. Inorganic Molten Salts

Inorganic molten salts are ionic liquids composed of one or more inorganic salts. They form a homogeneous liquid phase when heated above their melting point. They offer advantages such as good thermal stability, low vapor pressure, recyclability and environmental friendliness [37,38]. In a high-temperature molten state, molten salt provides a homogeneous reaction environment for metal precursors, promoting the controlled nucleation and growth of nanoparticles. It also serves as a non-toxic structural directing agent to regulate the formation of highly active crystal faces and specific nanostructures. This approach significantly simplifies the post-treatment process, aligns with the principles of green synthesis, and provides a viable pathway for the large-scale preparation of high-performance catalysts with low platinum loading [39,40].

The Zhao team [41,42,43] has conducted a series of systematic studies on the preparation of platinum-based catalysts in molten salt systems. In 2012, Zhao et al. [41] generated Pt^0^ and ammonia bubbles through the thermal decomposition of Pt(NH_3_)_4_C_2_O_4_ under alkaline conditions in freshly prepared or recycled KNO_3_-LiNO_3_ mixed molten salts. The platinum deposited at the gas and liquid interface and aggregated to form a continuous metal shell through Ostwald ripening, eventually yielding Pt concave nanoparticles. This method did not require the use of organic solvents or surfactants, and the resulting catalyst possessed an ultra-clean surface with exposed steps and high-index facets. Its specific activity (SA) for methanol oxidation reached 3.3 times that of commercial Pt/C, and its CO tolerance was significantly improved. Notably, the molten salt could be recycled four times without affecting the morphology or size of the products, demonstrating excellent reusability.

**Figure 4:**
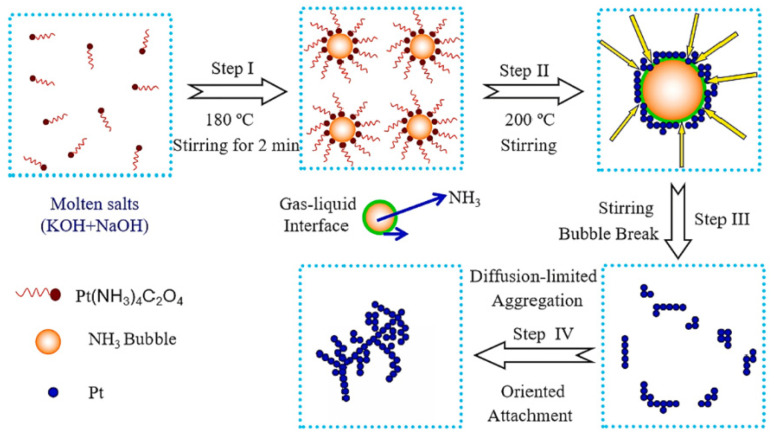
Schematic diagram of the formation process of Pt nanoflowers [42]. Copyright 2023, Elsevier.

**Figure 5:**
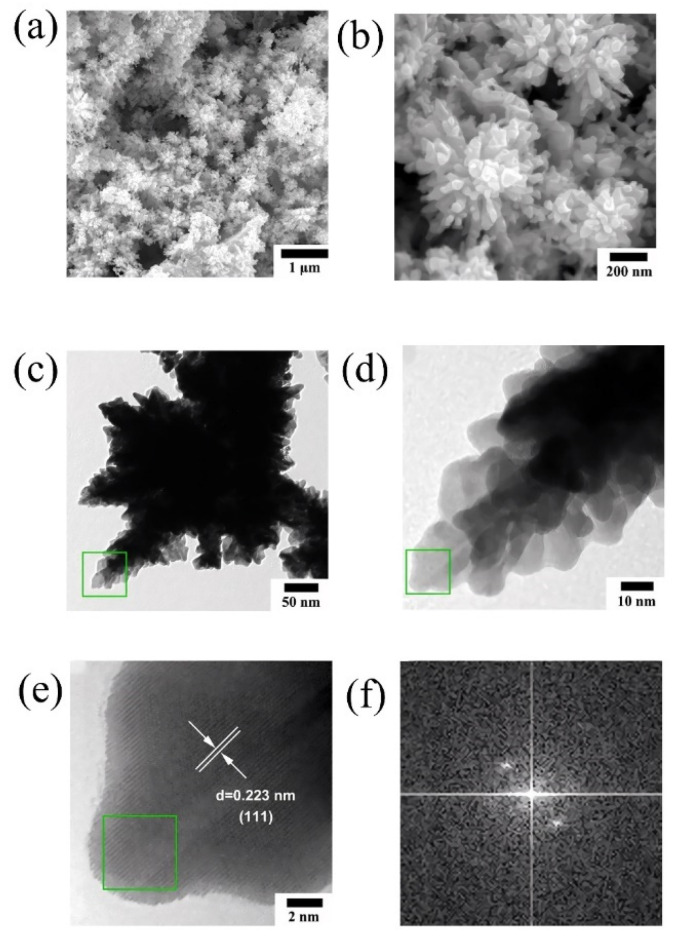
(**a**,**b**) Scanning Electron Microscope (SEM) images of Pt nanoflowers, (**b**) is the magnified image of (**a**). (**c**,**d**) TEM images of platinum nanoflowers; (**e**) High-Resolution Transmission Electron Microscopy (HRTEM) image of platinum nanoflowers; (**f**) Selected area electron diffraction Fourier transform image of the area labelled with a square in (**e**). Image (**d**) is the magnification of the area labelled with a square in (**c**); image (**e**) is the HRTEM of the area labelled with a square in (**d**) [42]. Copyright 2023, Elsevier.

Building on this work, Zhao et al. [42] extended the research system to prepare self-supported Pt nanoflower catalysts rich using NaOH and KOH as a low temperature molten salt medium. Figure 4 showed the schematic diagram of the formation process of Pt nanoflowers. Figure 5a,b intuitively confirmed that the product had a uniform flower-like structure with a particle size of approximately 400 to 500 nm, exhibiting a 3D self-supporting morphology without support agglomeration. Meanwhile, Figure 5c,d revealed the internal structure of the nanoflowers, which consisted of a central stamen and branched petals; the petals were formed by numerous nanosheets growing along the branch direction, further explaining the structural characteristics of a loose framework and high specific surface area. Figure 5e showed an interplanar spacing of 0.223 nm, corresponding to the (111) crystal plane of the face-centered cubic structure of Pt. Figure 5f demonstrated that the individual nanosheets constituting the nanoflowers exhibited a single-crystal growth mode, with highly consistent crystal orientation and strong structural stability. The methanol oxidation peak potential of this catalyst shifted negatively by 20 mV compared to commercial Pt black, and its long-term stability was significantly better than that of commercial Pt black. This self-supporting structure effectively avoided the issue of support corrosion in acidic media. In 2024, the same team [43] combined microwave and molten salt methods. Using KNO_3_ and LiNO_3_ molten salt, they shortened the reaction time to 15 min and rapidly and successfully synthesized platinum nanoparticles with a size of less than 50 nm. The as-prepared Pt concave nanoparticles exhibited a twin structure and a concave morphology. Their MA for formic acid oxidation reached 502.00 mA/mg_Pt_, which was approximately 4.6 times that of commercial Pt/C. Chronoamperometry (CA) tests showed that after 24 h of continuous testing, the MA of the catalyst remained about 4.5 times higher than that of commercial Pt/C, demonstrating excellent long-term stability and resistance to CO poisoning.

The main advantages of the molten salt method lie in its ability to efficiently prepare catalysts with special morphologies and ordered alloy structures. Additionally, the molten salt can be recycled, aligning with the principles of green synthesis. However, the corrosive environment of high-temperature molten salts hinders the application of in situ characterization techniques. Residues of molten salt ions may remain on the product surface, affecting the exposure of catalytically active sites. Furthermore, the energy consumption associated with process scale-up requires further optimization.

#### 2.1.4. Deep Eutectic Solvents

Deep eutectic solvents (DES) are a class of ionic liquids formed by mixing hydrogen bond donors and hydrogen bond acceptors in specific ratios, with a melting point significantly lower than the individual melting points of their components [44,45]. Due to their unique properties, such as low volatility, low toxicity, non-flammability, high chemical stability, low cost, and strong environmental compatibility, DES have become ideal media for green synthesis [46,47]. In the preparation of nanomaterials, DES not only provides a high-solubility environment for precursors, but can also act as morphology-directing agents and reducing agents. Their ionic nature helps inhibit the growth and agglomeration of nanoparticles through electrostatic stabilization, while their low interfacial tension facilitates the reduction in nucleation barriers, promoting the formation of small, well-dispersed particles [48,49].

In the application of DES systems, Wang et al. [50] used choline chloride and ethylene glycol as the reaction medium and reducing agent, respectively, through the synergistic regulation of PVP and SDS, hollow Pt nanoparticles with an open structure assembled by fine Pt grains were synthesized, which featured internal cavities and a rough surface. This unique structure fully exposed active sites, significantly enhancing methanol oxidation performance. The ECSA reached 24.7 m^2^/g_Pt_, which was 3.68 times that of commercial Pt black, the MA and SA were 5.4 times and 1.5 times that of commercial Pt black, respectively, with an onset potential as low as 0.26 V. After a 7200-s current decay test, its activity remained 4.65 times that of commercial Pt black. CO stripping tests indicated a more negative oxidation onset potential, demonstrating outstanding resistance to poisoning. Li et al. [51] used ethylene glycol and choline chloride as solvents and shape-directing agents, introduced PDDA to assist octahedral morphology formation, and combined the strong anchoring effect of Co–N co-doped carbon carriers with a flow-through microreactor to achieve the one-step in situ synthesis of PtPd nanocrystals in just 6 min. The resulting nanocrystals exhibited a uniform octahedral morphology with predominantly exposed (111) highly active facets. The nanocrystals achieved the MA of 201 mA/mg for the MOR, the ECSA of 26.4 m^2^/g, and an *I_f_*/*I_b_* ratio of 1.77, which was more than three times higher than that of commercial Pt/C. After a 3600-s CA test, they maintained relatively high activity, demonstrating excellent stability. PDDA is Poly (diallyldimethylammonium chloride).

The main advantages of the DES synthesis method lie in its mild reaction conditions and high functional integration, which avoid the need for high-temperature and high-pressure equipment. However, the high viscosity of DES limits mass transfer efficiency and electrical conductivity. Its stability in strongly acidic environments is insufficient. Both synthesis efficiency and batch-to-batch reproducibility require improvement. Additionally, its compatibility with fuel cell components needs further optimization.

### 2.2. Biosynthesis Systems

In recent years, green synthesis strategies, particularly biosynthesis, have emerged as a highly promising approach for preparing platinum nanoparticles for fuel cell applications. This method utilizes biomolecules derived from plant extracts, microorganisms, or their metabolites as green reducing agents, stabilizers, and capping agents. It efficiently reduces platinum precursors into nanoscale particles under mild conditions, such as ambient temperature and pressure and near-neutral pH [52,53,54]. Functional groups in these biomolecules interact with the surface of platinum nanoparticles to effectively suppress particle agglomeration while also enabling precise control over particle size, morphology, and dispersion through the adjustment of reaction parameters [53,55]. Compared to traditional chemical synthesis methods, the bio-reduction method avoids the use of toxic reagents and offers significant advantages such as mild reaction conditions, simple operation, renewable raw materials, and good environmental compatibility [56,57].

#### 2.2.1. Phytosynthesis

Phytosynthesis is one of the most extensively studied approaches within biosynthesis systems. Its core principle involves utilizing the bioactive components abundant in plant extracts (such as phenols, flavonoids, polysaccharides, organic acids, and proteins) to simultaneously achieve the reduction of platinum ions and the directed assembly of nanoparticles. This process requires no additional chemical reagents, thereby reducing the environmental burden at the source of synthesis [58,59]. In addition, plant resources are abundant and easily accessible, the preparation process is simple, and the method is readily scalable. More importantly, the carbon-based and oxygen-based phytochemicals in plant extracts can optimize the surface electronic structure of platinum nanoparticles through bioconjugation, significantly enhancing catalytic reaction kinetics [60,61].

Beygisangchin et al. [62] used sugarcane bagasse extract as a green reducing and stabilizing agent to synthesize Pt-CQD composite catalysts via a one-pot aqueous-phase green synthesis method. When the CQD (carbon quantum dots) concentration was 300 ppm, the obtained Pt-CQD300 catalyst had a particle size of approximately 4.8 nm, exhibiting optimal structure and dispersion. The catalyst achieved an ECSA of 98.87 m^2^/g. In the MOR, its maximum peak current density reached 138.48 mA/cm^2^, and its MA reached 688.25 mA/mg, and demonstrated excellent CO poisoning resistance as well as good cycling stability. Electrochemical impedance spectroscopy analysis indicated that this catalyst possessed a low charge transfer resistance, with improved conductivity and durability. However, excessive CQD loading led to increased resistance and reduced performance. Cruz et al. [63] used guarana extract as a green reducing agent to prepare platinum nanocatalysts supported on chitosan (PtNPs@Q) and activated carbon (PtNPs@C), respectively. The Pt nanoparticles in both catalysts had a particle size of 1–2 nm, but the Pt nanoparticles in PtNPs@C exhibited better dispersion without obvious aggregation due to the high specific surface area and conductivity of activated carbon. Attributed to the synergistic effect between the support and the active component, PtNPs@C exhibited superior catalytic activity and cycling stability in the ethanol oxidation reaction. In CA tests over 1800 s, its steady-state current was approximately four times higher than that of PtNPs@Q.

However, the composition of plant extracts is complex and subject to significant batch-to-batch variation, making precise control over the size and morphology of platinum particles difficult. Residual biomass can easily cover active sites, resulting in catalytic performance that is generally inferior to that of commercial Pt/C. In addition, the synthesis process suffers from poor reproducibility and low reaction efficiency, which hinders its ability to meet the requirements of industrial production in terms of batch-to-batch consistency and scalable manufacturing.

#### 2.2.2. Microbial Synthesis

The microbial synthesis method utilizes the intracellular enzyme systems or extracellular metabolites of microorganisms such as bacteria, fungi, and algae to achieve the reduction of platinum ions, the nucleation and growth of nanoparticles, and their in situ stabilization [64,65]. This process does not require the addition of external chemical reducing agents and offers key advantages such as mild reaction conditions, simple operation, low energy consumption, low pollution, and renewable resources, providing an important pathway for the preparation of green catalysts [66,67].

**Figure 6:**
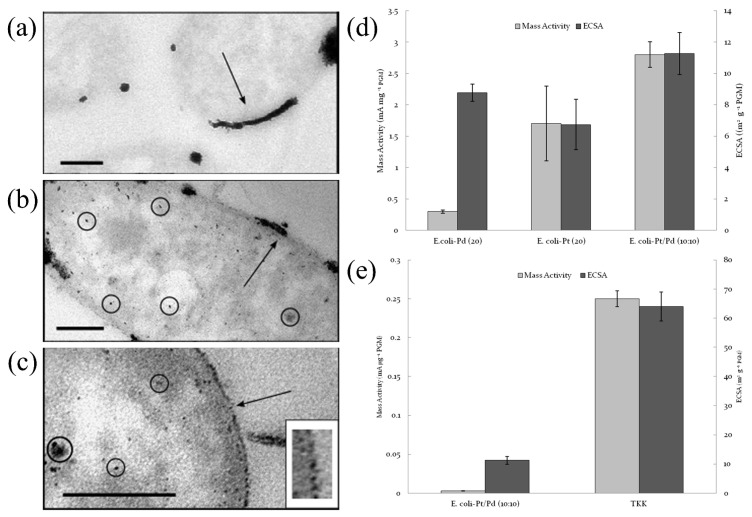
(**a**) TEM image of E. coli-Pt (10 wt.%), where agglomerated periplasmic Pt NPs are identified with a black arrow; note lack of intracellular NPs using equimolar Pt. (**b**,**c**) TEM images of bimetallic E. coli-Pt/Pd (10 wt.%: 10 wt.%), where the black arrow shows a degree of agglomeration at the periplasm, while intracellular nanoparticles can be seen (circled). The higher magnification image (**c**) shows a “nanoparticle shell” in the periplasm as indicated by the black arrow. Bars are 200 nm. Inset: expanded region of the cell surface of the cell shown in (**c**), showing discrete supported NPs. (**d**) ECSA and mass activity of E. coli-Pd (20%), E. coli-Pt (20%), and E. coli-Pt/Pd (10%:10%). (**e**) Comparison of ORR mass activity and ECSA between biosynthesized E. coli-Pt/Pd (10%:10%) and commercial TKK catalyst [68]. Copyright 2019, Frontiers Media SA.

Current research mainly focuses on the innovation and performance optimization of microbial synthesis strategies. Stephen et al. [68] reported bimetallic Pt/Pd nanoparticles (NPs) biosynthesized by E. coli [E. coli-Pt/Pd (10 wt.%:10 wt.%)]. Figure 6a showed agglomerated Pt nanoparticles (black arrow) on the surface of E. coli-Pt (10%); Figure 6b,c showed that the cell surface of the bimetallic E. coli-Pt/Pd (10 wt.%:10 wt.%) also exhibited nanoparticle protrusions that formed a “shell”, demonstrating that agglomerated nanoparticle protrusions were observed on the cell surface, forming catalytically active sites. The electrocatalytic tests in Figure 6d indicated that the MA and the ECSA of E. coli-Pt/Pd (10%:10%) were superior to those of E. coli-Pt (20 wt.%) and E. coli-Pd (20 wt.%). Although limited by the inherent resistance of the bacteria and the internal localization of the nanoparticles, the biosynthesized PtPd bimetallic catalyst (without optimization) contained only about one-fifth the platinum content of a commercial PEMFC catalyst, and its ORR performance was approximately 100 times poorer (Figure 6e). Nevertheless, this green synthesis strategy provided an important starting point for the engineered biosynthesis of PEMFC catalysts.

The advantage of microbial synthesis lies in its excellent biocompatibility and great potential for structural regulation. However, it faces challenges such as stringent culture conditions, high costs, long growth cycles, low yields, and significant batch-to-batch variations in nanoparticle size and morphology, which limit its large-scale application.

### 2.3. Renewable Resource Templates

Renewable resource templates have emerged as a key direction in the development of platinum-based fuel cell catalyst supports, thanks to their dual advantages of environmental sustainability and structural tunability. Derived from biomass, biomacromolecules, or natural minerals, these templates can be used to construct carbon-based and inorganic supports with high specific surface areas, abundant active sites, and unique confined structures through processes such as pyrolysis, self-assembly, or in situ modification. This approach not only reduces the environmental impact of catalyst preparation but also optimizes the dispersion, stability, and catalytic kinetics of nanoparticles through metal-support interactions.

#### 2.3.1. Biomass-Derived Carbon Supports

Biomass-derived carbon supports are prepared into porous carbon materials through high-temperature pyrolysis and activation treatments, which preserve their natural hierarchical structure and pore characteristics, thereby facilitating mass transfer and exposing active sites [69,70,71]. Biomass is rich in components such as proteins and amino acids, enabling self-doping of elements including nitrogen, phosphorus, and sulfur. Among these, nitrogen doping effectively modulates the electronic structure of carbon, enhances electrical conductivity, and provides active sites, thereby forming a synergistic catalytic effect with metal sites [71,72].

Medellín et al. [73] proposed a novel green preparation route. In this method, Sargassum extract was used as a reducing agent to green-synthesize platinum nanoparticles with particle sizes of 3 to 7 nm, which were then loaded onto two types of carbon supports: one was Vulcan XC-72, and the other was SKPH (derived from the pyrolysis of the same Sargassum biomass, with a SA as high as 2289 m^2^/g). FTIR analysis identified hydroxyl groups and polysaccharide sulfates as the key functional groups responsible for the reduction of Pt^4+^. The resulting 20% SPtNPsp-SKPH electrocatalyst exhibited a current density of 30 mA/cm^2^ in CV tests, along with excellent ORR activity in acidic media, featuring an onset potential close to 0.9 V and an electron transfer number of 4.2. The work by Kim et al. [74] achieved a breakthrough in the study of support functionalization. They developed a silicon carbide-modified graphene-supported platinum catalyst (SiC/G@Pt composite catalyst) using coffee grounds as the carbon source for epitaxial graphene growth. This study not only addressed the poor conductivity of pure SiC but also provided abundant anchoring sites for Pt. The synergistic interaction between the graphene modification layer and the SiC substrate formed a strong metal-support interaction to stabilize the Pt nanoparticles. Furthermore, leveraging the excellent chemical and mechanical stability of SiC, this study significantly suppressed carbon corrosion and Pt agglomeration during electrochemical cycling, thereby greatly enhancing the catalyst durability.

The key advantages of biomass-derived carbon supports lie in the low cost and wide availability of raw materials, as well as the ability to recycle waste into valuable resources. However, significant challenges remain: substantial batch-to-batch variations in biomass composition result in poor structural uniformity of the supports; amorphous carbon inherently lacks sufficient conductivity and typically requires doping and modification; and residual ash from the pyrolysis process may cover active sites, thereby reducing catalytic performance. These issues can be further addressed through strategies such as feedstock pretreatment, controlled doping, and high-temperature purification.

#### 2.3.2. Biological Structure Templating Method

In recent years, biomacromolecules such as peptides, proteins, and nucleic acids have become ideal biological scaffolds for synthesizing nanomaterials due to their unique self-assembly capabilities and structural diversity [75,76]. The biostructural templating method utilizes the ordered structures formed by their self-assembly as templates, enabling precise control over the nucleation, growth, and spatial arrangement of metal nanoparticles. It is particularly suitable for the green and controlled preparation of platinum-based catalysts for fuel cells [77]. This method can guide the uniform dispersion and oriented growth of Pt particles within multi-level pore channels and confined spaces, achieving fine-scale structural assembly that is difficult to realize using traditional chemical methods.

Hou et al. [76] used insulin amyloid fibrils (INSAFs) as a template to prepare vine-tree-like PtRh alloy nanocatalysts (VT-PtRhNWs). In this structure, the inner PtRh nanowires served as the “trunk,” while the outer PtRh nanoparticles interconnected to form a spiral chain-like structure resembling flexible “vines.” Both the nanoparticles and nanowires had sizes below 2 nm, and the ECSA reached 79.20 m^2^/g. Its MA for the MOR reached 455 mA/g, which was 1.86 times that of commercial Pt/C, with significantly enhanced CO tolerance and cycling stability. Ponjavic et al. [78] used bacterial nanocellulose (BNC) as a green template to synthesize a Pt/BNC catalyst via a microwave-assisted polyol method. The abundant hydroxyl and carboxyl functional groups on the BNC surface, together with the reducing and stabilizing effects of ethylene glycol, enabled highly uniform dispersion of Pt nanoparticles on the template surface. In MOR tests, the as-prepared catalyst exhibited a significantly negative-shifted onset potential compared with commercial Pt/C ETEK, a peak current density of 0.82 mA/cm^2^, and a CO tolerance. It also retained good activity after 100 cycles and showed a gradual current decay over 1800 s in CA testing. These results confirmed the enhancing effect of the biological template on catalytic stability by inhibiting Pt agglomeration and CO poisoning.

**Figure 7:**
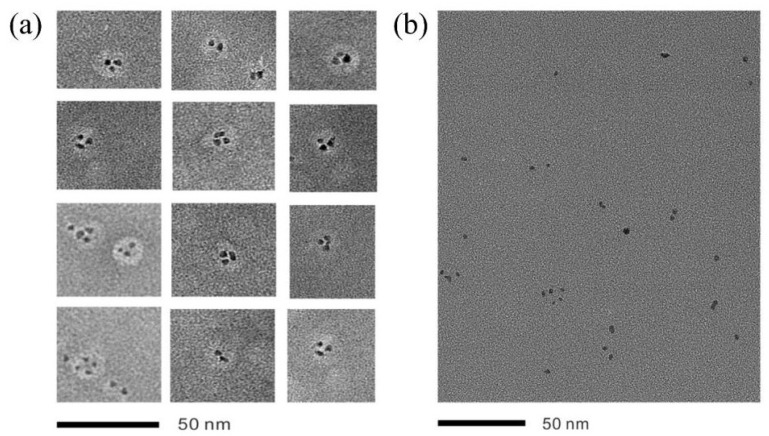
Representative TEM images (negatively stained) (**a**) Pt NR, (**b**) Pt NP [79]. Copyright 2021, American Chemical Society.

The work by Pan et al. [79] took precision to a new level. By exploiting the reversible self-assembly behavior of tobacco mosaic virus coat protein (TMVCP) at different pH levels, they successfully synthesized platinum nanorings (Pt NR) and platinum nanoparticles (Pt NP). Figure 7 showed TEM images of Pt NRs and Pt NPs, in which Pt NRs were assembled into closely packed ring structures on the protein disk template, while Pt NPs were in an isolated, dispersed state on the protein template. In particular, the interparticle spacing of the platinum nanorings was narrow, approximately 0.94 nm. This highly uniform spatial arrangement induced a strong interparticle synergistic effect, which significantly enhanced charge transfer kinetics and intrinsic catalytic activity. This work demonstrated the unique advantage of biological templates in constructing fine nanostructures, which was difficult to achieve with traditional chemical methods.

The main advantage of the biological structure templating method lies in its ability to achieve fine structural regulation that is difficult to realize with conventional chemical methods. However, biological templates, especially proteins, suffer from poor structural stability under harsh operating conditions, making them prone to denaturation and degradation. Additionally, their extraction and purification are costly, and scale-up production remains challenging.

### 2.4. Energy-Saving Methods

Traditional methods for preparing platinum catalysts often rely on high-temperature calcination, prolonged reaction times, and energy-intensive equipment. These methods suffer from limitations such as waste resulting from precious metal agglomeration, significant environmental impact, and high production costs, which hinder the large-scale application of the technology and are inconsistent with the principles of sustainable development [80,81]. Green and energy-saving methods introduce non-traditional energy inputs, such as microwaves, light, electricity, mechanical force, and plasma, to revolutionize reaction pathways. These approaches enhance synthesis efficiency and reduce energy consumption while achieving precise and clean construction of platinum nanostructures [82,83]. The core commonality of these methods lies in the precise intervention of external energy fields to accelerate reactions, suppress byproducts, and regulate product characteristics.

#### 2.4.1. Microwave Method

The microwave method, leveraging its characteristics of instantaneous polarization and rapid, uniform heating, can achieve rapid reduction of platinum precursors and uniform nucleation and growth of nanoparticles within an extremely short time, thereby significantly reducing both energy consumption and reaction time [80,84]. Chen et al. [84] reported that microwave-assisted synthesis significantly increased the reaction rate and greatly accelerated the reduction and nucleation of metal ions. Consequently, microwave heating can effectively control the size, morphology, and crystal orientation of platinum-based catalysts, resulting in a more uniform nanoparticle size distribution. This approach significantly enhances platinum atom utilization and catalytic activity while making the preparation process more environmentally friendly [82].

Paez et al. [85] and Sharma et al. [86] demonstrated the efficiency advantages of the microwave method. They successfully synthesized highly dispersed carbon-supported platinum nanoparticles within minutes in aqueous or polyol systems, respectively, without the need for strong reducing agents. The rapid and uniform heating of microwaves promoted the instantaneous formation of a large number of uniform crystal nuclei, resulting in platinum particles with a small size and a narrow size distribution. Consequently, under extremely low platinum loading, they achieved an ECSA superior to or comparable to that of commercial catalysts. Jo et al. [87] developed a microwave plasma spray pyrolysis technique that enabled the one-step synthesis of Pt/GR composites in just 0.74 s. Pt nanoparticles were uniformly deposited on the surface of wrinkled graphene. The resulting material achieved a SA of 402 m^2^/g and an ECSA of 77 m^2^/g. Its anti-poisoning ability and long-term stability were both superior to those of commercial Pt/C.

The advantages of this method lie in its fast reaction rate and uniform particle size. However, microwave heating is prone to localized overheating, ensuring uniform distribution of the microwave field in large-scale production is challenging, and the risk of particle agglomeration increases in precursor systems with high concentrations.

#### 2.4.2. Photoreduction Synthesis

Photoreduction synthesis utilizes light energy to excite photosensitizers or semiconductors, generating photo-generated electrons that can reduce metal precursors at room temperature or under mild conditions, making it a clean and efficient green preparation technique [88,89]. The green essence of this method lies in its use of photons as a clean energy source, avoiding the high-temperature, high-pressure conditions and strong chemical reducing agents required in conventional synthesis. Additionally, it allows effective control over the reduction kinetics by adjusting the illumination parameters [89]. Harada et al. [90,91,92,93] systematically studied the photoreduction process of Pt under UV light, discovering that rapid reduction steps were conducive to the formation of small-sized nanoparticles and elucidating the growth mechanism of these nanoparticles.

Badam et al. [94] first achieved the in situ generation of Pt nanoparticles on different conductive carbon substrates under simulated solar light, using TiO_2_ as the photocatalyst and water as the solvent, without any sacrificial reducing agents. This work revealed the critical influence of substrate conductivity on photoelectron diffusion and Pt nucleation. Building on this work, Bukka et al. [95] utilized the photogenerated electron spillover effect of TiO_2_ nanotubes to photoreduce H_2_PtCl_6_ into ultra-small Pt nanoparticles with an average particle size of 1.6 nm, with the assistance of formic acid. The Ti–C bonds formed between TiO_2_ nanotubes (TNT) and the functionalized acetylene black substrate (FAB) enhanced the metal-substrate interaction, enabling the catalyst to exhibit a MA of 37.5 A/g even at a low Pt loading of only 3.5 wt%. Moreover, the current retention rate remained as high as 95.9% after 2000 cycles, and its performance was significantly superior to that of commercial Pt/C catalysts.

The limitation of the photoreduction method lies in its efficiency being dependent on photosensitizers and photocatalysts, along with the limited light penetration depth, which makes it unsuitable for the efficient production of high-concentration and large-volume reaction systems. Furthermore, most current studies adopt ultraviolet light or simulated sunlight. Therefore, expanding the photoresponse range to the lower-energy and more abundant visible light region, and developing more stable and efficient photosensitive systems, are key directions for improving the generality and practicality of this technology.

#### 2.4.3. Electrodeposition Method

Electrochemical deposition is a reliable approach for the green synthesis of metal nanoalloy catalysts [96]. By precisely regulating the potential, current density, and deposition time under ambient temperature and pressure, the electrodeposition method enables the in situ construction of catalyst layers with specific morphologies, sizes, and crystal facets directly on gas diffusion layers or carbon supports. This realizes the integration of catalyst synthesis and electrode preparation, significantly improving the atomic utilization efficiency of platinum and the mass transfer efficiency of the electrode [97,98].

Zhang et al. [99] utilized the square-wave potential method to achieve controlled electro-deposition of platinum on carbon paper, yielding spherical, cauliflower-like, coral-like, and thorn-like structures, with the morphological complexity increasing significantly as the deposition potential became more negative. Among these, the cauliflower-like platinum, due to its high roughness and uniform dispersion, exhibited the ECSA as high as 83.3 m^2^/g_Pt_, which was 4.6 times that of commercial platinum black. Its MA for the MOR and ammonia oxidation reaction (AOR) was 4.7 times and 3.9 times that of commercial platinum black, respectively, and its current retention rate after 3600 s of stability testing reached 32.4%. Dhanasekaran et al. [100] achieved the structural evolution of Pt from nanospheres to three-dimensional nanoflowers via galvanostatic electrodeposition. The as-prepared catalyst exhibited a MA of 5500 mA/mg_Pt_ and a peak power density of 660 mW/cm^2^ in the H_2_-O_2_ system of PEMFCs. During the cumulative 33 h stability test, the current density decreased by only approximately 10%, demonstrating excellent stability.

However, this method still faces challenges in controlling the uniformity of large-scale preparation and achieving uniform deposition on non-planar electrodes. How to combine theoretical simulation with process regulation to realize the leap from laboratory research to scale-up production is an important direction for future studies.

#### 2.4.4. Mechanochemical Method

Mechanochemistry is an environmentally friendly and controllable catalyst preparation strategy that adheres to the principles of green chemistry [101,102]. Through the mechanical force generated by high-energy ball milling, this method induces physical fragmentation, chemical bond cleavage, and recombination of solid reactants at the interface, enabling the solid-phase synthesis and uniform loading of platinum-based catalysts. Its prominent advantage lies in minimizing solvent usage, thereby avoiding solvent pollution and subsequent recycling challenges from the source [83].

**Figure 8:**
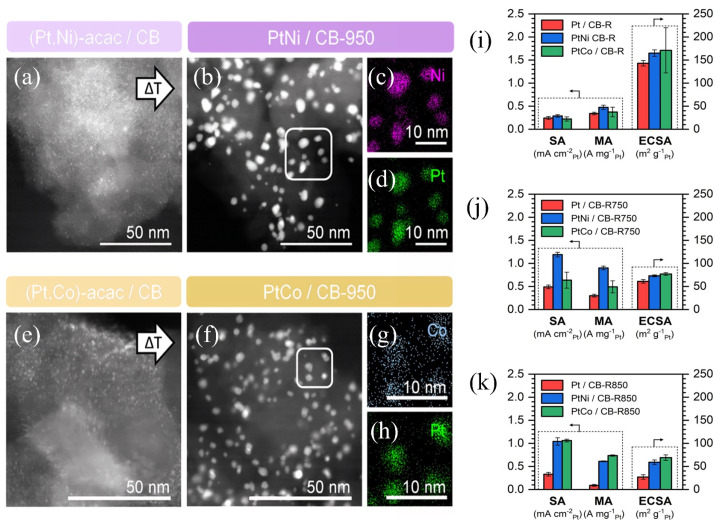
High-Angle Annular Dark-Field Scanning Transmission Electron Microscopy (HAADF-STEM micrographs) micrographs for (**a**) (Pt, Ni)-acac/CB and (**b**) PtNi/CB-950. (**c**) Ni-K and (**d**) Pt-L EDX elemental maps measured on PtNi/CB-950 in the area highlighted in panel (**b**). HAADF-STEM micrographs for (**e**) (Pt, Co)-acac/CB and (**f**) PtCo/CB-950. (**g**) Co-K and (**h**) Pt-L EDX elemental maps measured on PtCo/CB-950 in the area highlighted in panel (**f**). ORR activity, as SA and MA, and ECSA for 10 wt %Pt(M)/CB-RXXX (M = Ø, Ni, Co) obtained from (1 Pt, 1 M)-acac/CB after reduction at 220 °C (**i**) and reduction and annealing at 750 °C (**j**) or 850 °C (**k**) [104]. Copyright 2023, American Chemical Society.

Mukherjee et al. [103] ground and mixed Pt, Co, and Ni acetylacetonates with Ketjenblack carbon support, followed by annealing at 800 °C for 4 h under N_2_ atmosphere to synthesize the Pt_3−x_Co_0.5+y_N_0.5+y_/C (x = 0, 1, 2; y = 0, 0.5, 1) alloy catalyst. High-temperature annealing induced the transformation of the disordered face-centered cubic alloy to the ordered face-centered tetragonal L1_0_ phase, optimized the lattice order and the electronic structure of Pt, enhanced the synergistic effect among Pt, Co, and Ni components, and significantly improved the ORR activity and long-term stability of the catalyst. Gunnarson et al. [104] prepared PtNi and PtCo alloy nanoparticles by dispersing metal acetylacetonates and carbon supports via planetary ball milling, followed by H_2_ reduction at 220 °C and annealing under an argon atmosphere. As observed from Figure 8a,e, the metal species in the precursor were uniformly distributed on the surface of the carbon support after ball milling; after annealing, alloy nanoparticles with uniform sizes were formed without obvious agglomeration (Figure 8b,f). Among them, Pt was closely mixed with Ni or Co, as illustrated by the EDX elemental maps (Figure 8c,d,g,h). Specifically, PtNi/CB-R750 exhibited the ECSA of 70 m^2^/g_Pt_ and MA of 0.9 A/mg_Pt_ for the ORR (Figure 8j), which exceeded the catalytic activity target set by the U.S. Department of Energy and outperformed traditional commercial catalysts. After 10,800 potential cycles, the ECSA loss was less than 10%, demonstrating excellent stability.

Despite the promising application potential of the mechanochemical method, its large-scale application still faces key challenges: slight fluctuations in milling parameters such as ball-to-powder ratio, rotation speed, and milling time are prone to causing significant differences in product performance, making it difficult to precisely control preparation reproducibility; wear of the milling medium is likely to introduce impurity contamination; meanwhile, the ability to achieve nanoscale precise and directional regulation of product morphology and size remains relatively limited.

#### 2.4.5. Plasma Method

Plasma technology, especially low-temperature plasma processes, has gained extensive attention in materials science and energy fields due to its advantages of environmental friendliness, high efficiency, controllability, and minimal damage to material structures [105,106]. At near room temperature, high-energy active particles generated by this technology act on the surface of precursors or supports. This enables rapid reduction and uniform loading of nanoparticles. It avoids the use of toxic reagents, simplifies the process flow, and reduces impurity introduction. Meanwhile, it achieves precise regulation of microstructures that is difficult to attain with traditional chemical methods [107,108].

**Figure 9:**
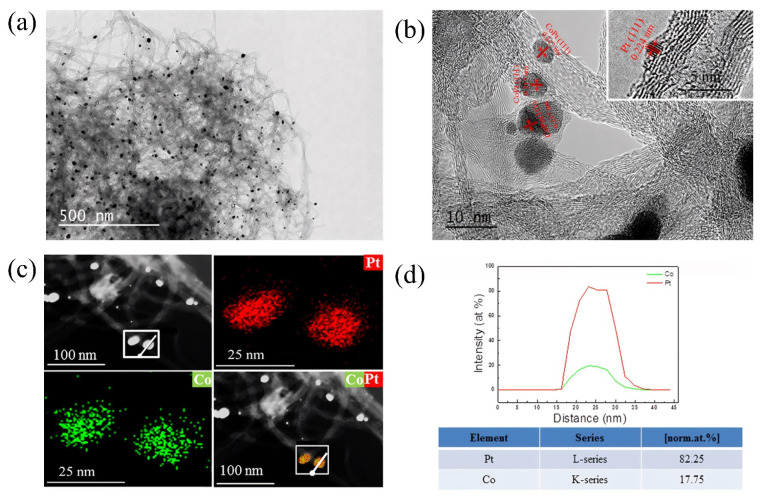
(**a**) TEM and (**b**) HRTEM images of Pt/CoPt-2/MWCNTs. The inset shows the HRTEM image of Pt NP. (**c**) HAADF image and EDS (Energy-Dispersive X-ray Spectroscopy) mapping of Pt, Co and the composite of Pt and Co. (**d**) Line and area profiles of Pt and Co extracted from the white line and box indicated in (**c**) [110]. Copyright 2017, Springer Nature.

**Figure 10:**
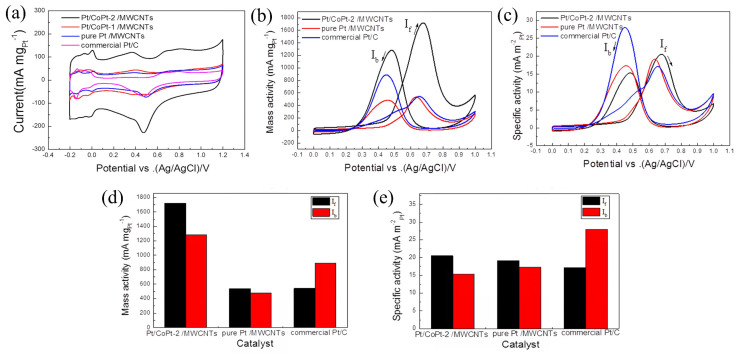
(**a**) CVs of Pt/CoPt-2/MWCNTs, Pt/CoPt-1/MWCNTs, pure Pt/MWCNTs and commercial Pt/C measured in argon-purged 0.5 M H_2_SO_4_ at a scan rate of 50 mV/s. (**b**) Mass activity and (**c**) specific activity of the three catalysts towards methanol oxidation along with respective bar graphs (**d**,**e**) measured in 0.5 M H_2_SO_4_ and 1 M MeOH at a scan rate of 50 mV/s [110]. Copyright 2017, Springer Nature.

Zha et al. [109] developed a two-step radio-frequency plasma treatment strategy. Before the start of the experiment, the pristine NCFs@CC had a smooth surface with few defects. After plasma treatment, p-NCFs@CC still maintained a slender tubular structure, but the outer wall became very rough. This was due to the etching of the NCFs surface by the plasma, creating abundant carbon defects and oxygen vacancies. At the same time, this strategy increased the specific surface area and oxygen-containing functional group content of the support, enhancing the anchoring and dispersion of PtNi nanoparticles. PtNi NPs were uniformly loaded on the surface of p-NCFs with an average particle size of 4.96 nm and no obvious agglomeration, confirming the anchoring effect of the first support modification. After the second plasma treatment, defects such as lattice distortion and atomic dislocation appeared inside the PtNi NPs. The second plasma treatment reduced the oxidation state of PtNi, increased the proportion of metallic Pt^0^, and introduced lattice defects. The strong interaction between the defective support and PtNi optimized the electronic structure. Density Functional Theory (DFT) calculations confirmed that the defects and low-valence PtNi introduced by plasma treatment significantly lowered the energy barriers of the rate-determining steps in ORR and MOR and weakened CO adsorption, thereby improving catalytic activity and anti-poisoning ability. Huang et al. [110] used solution plasma technology with Pt/Co wires as electrodes to synthesize Pt/CoPt-1 nanoparticles by discharge in deionized water. After compositing with Multi-Walled Carbon Nanotubes (MWCNTs), it was annealed to form highly alloyed Pt/CoPt-2/MWCNTs. Figure 9 showed that the nanoparticles were uniformly dispersed with clean surfaces, and the lattice fringes of the CoPt alloy were clearly resolved. The ECSA of this catalyst reached 83.7 m^2^/g (Figure 10a), which was much higher than those of pure Pt/MWCNTs and commercial Pt/C. Benefiting from the clean surface, high specific surface area, and synergistic effect of the CoPt alloy, its MA for the MOR reached 1719 mA/mg, which was 3.16 times that of commercial Pt/C, and its electrocatalytic activity, CO poisoning resistance, and stability were significantly enhanced (Figure 10c,d).

Although low-temperature plasma technology has shown remarkable advantages at the laboratory scale, its industrialization still faces prominent bottlenecks: the reaction of high-energy species is highly random, making it difficult to precisely control the platinum particle size, defect concentration, and support etching depth; the discharge uniformity is insufficient in large-scale production; moreover, the high equipment investment and operating energy consumption restrict its large-scale application.

#### 2.4.6. Rapid Joule Heating Method

Rapid Joule heating is a novel catalyst preparation technology integrating the advantages of high efficiency, environmental friendliness, and precise controllability. Relying on instantaneous high-temperature heat treatment, this method enables rapid thermal reduction and alloying of metal precursors within a millisecond time scale. It requires no additional solvents or reducing agents, thereby avoiding the use of organic reagents and additives in traditional methods, significantly simplifying the process flow and reducing environmental pollution [111]. Meanwhile, Joule heating can achieve uniform dispersion of nanoparticles and precise structural regulation on mild substrates. This effectively enhances the electrochemical activity and long-term stability of the catalyst, providing a new approach for the green synthesis, low-cost preparation, and large-scale production of fuel cell electrode materials [112,113].

Deng et al. [114] synthesized uniformly dispersed PtRu alloy nanoparticles with a narrow particle size distribution on carbon black via direct Joule heating. The TEM images showed that under the optimal conditions of 1000 °C and 50 ms, the obtained PtRu nanoparticles were smaller and more uniform. The particle size distribution data confirmed that the average particle size of PtRu/C-HT was 6.5 nm, while that of PtRu/C-JH-1000-50 was only 2 nm with the narrowest distribution. PtRu/C-JH-1000-50 exhibited an ECSA of 239 m^2^/g, and its MA for methanol oxidation was 2.8 times that of commercial Pt/C. In fuel cell tests, it retained 85.3% of its current density after 24 h, demonstrating excellent stability. DFT calculations confirmed that the Pt sites in the PtRu alloy exhibited strong methanol adsorption and weak CO adsorption, whereas pure Ru suffered from excessively strong CO adsorption and pure Pt was prone to CO poisoning. To further address the bottleneck of insufficient activity of single-atom Pt in the MOR, Lui et al. [115] synthesized a high-entropy alloyed single-atom Pt catalyst (Pt_1_-NiCoMgBiSn) in situ on carbon nanotube films via Joule heating. Through the synergistic regulation of the electronic structure of single-atom Pt sites by multi-element coordination, the high-entropy structure not only avoided the CO formation pathway to achieve anti-CO poisoning, but also promoted the direct oxidation of methanol. It exhibited excellent anti-CO poisoning performance and high methanol oxidation activity, with a MA of 35.3 A/mg_Pt_ under alkaline conditions.

Although Joule heating is efficient and environmentally friendly, it still has obvious shortcomings: the instantaneous high temperature and millisecond-scale reaction make it difficult to control the uniformity of the temperature field and composition, easily leading to particle size fluctuations; the specialized equipment and high energy consumption are not conducive to large-scale production; carbon supports are prone to overheating and oxidation, which affects the structure and stability of the catalyst; in addition, it relies on high-conductivity substrates, limiting the universality of the supports.

### 2.5. Performance Comparison and Rational Design of Catalysts

#### 2.5.1. Performance and Sustainability Comparison Between Green and Non-Green Synthesis

As can be seen from the comparison of the performance of various fuel cell catalysts listed in Table 1, there are significant differences between green and non-green synthetic routes in terms of platinum loading level, ECSA, electrochemical activity, and sustainability, clearly reflecting the inevitable trend in the current electrocatalysis field transitioning from high platinum dependence to low-platinum/platinum-free catalysts, and from traditional non-green processes to green, mild synthesis.

Although green synthesis strategies have made significant progress in recent years, a comprehensive assessment of their techno-economic competitiveness still requires a comparison of their key performance with that of traditional non-green preparation methods. Wang et al. [120] employed a vacuum impregnation method to confine Pt_1_Ru_3_ atomic alloys within mesoporous carbon hollow spheres (MCHS), followed by heat treatment at 800 °C, yielding the Pt_1_Ru_3_@MCHS catalyst. During the preparation, toxic HF was used to remove the silica template, large amounts of organic solvents (e.g., ethanol, formaldehyde) were consumed, and a prolonged high-temperature carbonization step with high energy input was involved, making the overall process clearly environmentally unfriendly. Although this catalyst exhibited an exceptionally high mass activity (5860 mA/mg_Pt_) toward the MOR under alkaline conditions, far superior to that of commercial Pt/C, its non-green synthetic route limited its potential for sustainable application. Qin et al. [118] successfully prepared a highly dense and well-ordered L1_0_-PtCo intermetallic catalyst supported on hollow mesoporous carbon (HMC) using a ligand-controlled co-reduction and high-temperature pyrolysis strategy. In contrast, the green synthetic routes summarized in this review achieved ORR/MOR/FAOR performance comparable to or even approaching commercial standards with lower platinum loadings, milder conditions, and more environmentally friendly processes, offering irreplaceable advantages in cost-effectiveness, stability, and poison tolerance.

#### 2.5.2. Strategies for Platinum Loading Regulation and Comparison with Palladium and Other Precious Metals

Regarding the developmental strategies for platinum mass loading, the data in Table 1 intuitively demonstrates that the electrocatalysis field was undergoing an accelerated transition from high-Pt loading to ultra-low-Pt loading.

High-Pt loading systems (Pt-CQD300 with a platinum loading as high as 82.5 wt%) could achieve relatively high initial mass activity, but suffered from high cost, low atomic utilization efficiency, and large resource consumption, which seriously violated the concepts of sustainable and large-scale preparation. In contrast, reducing platinum loading and improving atomic utilization efficiency have become the core development directions of green electrocatalysis. As shown in Table 1, green synthesis strategies such as molten salt method, phytosynthesis, microwave method, and mechanochemical method could effectively control platinum loading within the low range of 2.6 to 20 wt%. Among them, PtRu/C-JH-1000-50 exhibited an ultrahigh ECSA of 239 m^2^/g, and PtFe/B-FeNC achieved a MA of up to 2570 mA/mg, simultaneously enhancing catalytic performance and noble metal utilization while significantly reducing platinum consumption. Although current green synthesis could achieve efficient catalysis with low platinum loading, the actual platinum loading in industrial-scale production remained generally high. This was because the mass transfer efficiency, long-term stability, and poisoning resistance of low-platinum catalysts in membrane electrode assemblies could not yet fully meet the demands of industrial applications. Therefore, further reducing platinum loading while maintaining high activity and stability under practical working conditions has remained an urgent challenge to be overcome in green electrocatalysis. Furthermore, palladium-based and non-noble metal catalysts offered a feasible pathway for transitioning from low-platinum to platinum-free catalysts.

Compared with platinum, palladium has become an important alternative material in the field of fuel cell catalysts due to its abundant reserves and low cost. Zhu et al. [121] used wheat flour as a precursor and employed alkali-assisted pyrolysis combined with a template-free solvothermal method to in situ grow an interconnected Pd nanowire network on a nitrogen-doped porous carbon skeleton, denoted as Pd/NPC. The catalyst achieved a MA of 2818 mA/mg for alkaline methanol oxidation and 1582 mA/mg for acidic formic acid oxidation, with an ECSA as high as 142.1 m^2^/g. After a 5000 s stability test, it still maintained high activity and low charge transfer resistance, comprehensively outperforming conventional Pd/C, Pd/CNT, and Pd/RGO catalysts. Liu et al. [122] employed electrochemically active bacteria as both a bioreducing agent and an in situ doping source, combined with graphene oxide coating and hydrothermal carbonization, to prepare a three-dimensional porous heteroatom-doped PdAu/rGO electrocatalyst (DPARH). The catalyst exhibited excellent bifunctional activity toward alkaline ethanol oxidation and acidic formic acid oxidation, achieving MA of 8.30 A/mg and 2.04 A/mg, respectively, which were 6.15 and 6.58 times those of commercial Pd/C. Moreover, its current decay was significantly slower in a 2000 s stability test, and its overall performance surpassed that of commercial Pd/C and most previously reported palladium-based catalysts.

The above studies indicated that palladium-based catalysts exhibited high activity and good poison tolerance under alkaline conditions, making them the most promising alternatives to platinum-based catalysts. However, due to limitations such as poor stability in acidic environments and relatively slow intrinsic catalytic kinetics, palladium-based catalysts are still unable to fully replace platinum in fuel cells at present. In addition, ruthenium, iridium, rhodium, and other such metals are often used as alloying elements to composite with palladium or platinum, which can further enhance poison tolerance and activity by modulating the electronic structure and d-band center. However, these metals are scarce and expensive, and can only serve as synergistic modifying components rather than replacing the dominant role of platinum or palladium. Future research may further explore multinary noble metal-non-noble metal composite systems as well as non-noble metal oxides, carbides, and nitrides, aiming to maintain or even enhance catalytic performance while reducing overall noble metal loading through atomic-scale interface engineering and synergistic effects.

#### 2.5.3. Revelation of Catalytic Mechanism and Rational Design of Active Sites Based on Theoretical Calculations

In addition to experimental performance comparisons and the development of green synthesis strategies, theoretical calculations play an irreplaceable role in revealing catalytic mechanisms and guiding the rational design of catalysts.

**Figure 11:**
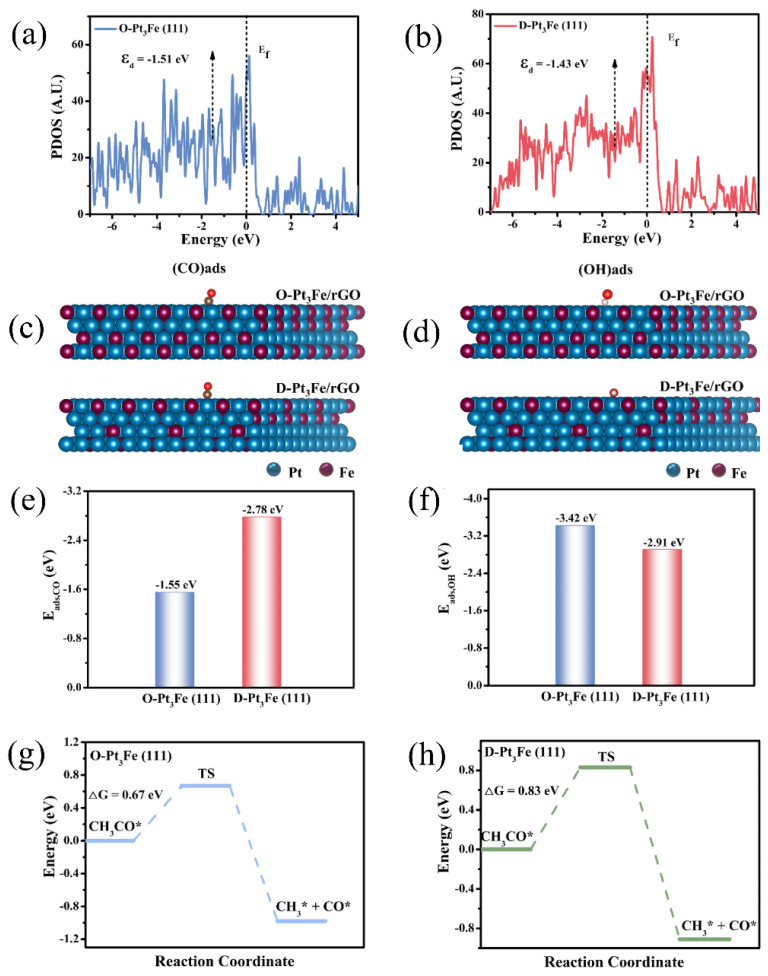
(**a**,**b**) PDOS analysis of the Pt d-band of O-Pt_3_Fe and D-Pt_3_Fe; (**c**,**d**) atomic structures (side view) for CO adsorption on O-Pt_3_Fe (111) and D-Pt_3_Fe (111) and the corresponding CO adsorption energies; (**e**,**f**) atomic structures (side view) for OH adsorption on O-Pt_3_Fe (111) and D-Pt_3_Fe (111); (**g**,**h**) DFT-calculated reaction energy barriers for C–C bond scission on the (111) facets of O-Pt_3_Fe and D-Pt_3_Fe [112]. Copyright 2024, American Chemical Society.

Nie et al. [112] employed an ultrafast Joule heating method to prepare ordered Pt_3_Fe intermetallic electrocatalysts supported on reduced graphene oxide (O-Pt_3_Fe/rGO). DFT calculations were performed to systematically compare the electronic structures and ethanol oxidation reaction (EOR) mechanisms of the ordered (O) and disordered (D) Pt_3_Fe (111) facets. PDOS analysis (Figure 11a,b) revealed that the Pt d-band center of O-Pt_3_Fe (−1.51 eV) was significantly lower than that of D-Pt_3_Fe (−1.43 eV), which was attributed to the larger lattice strain of the ordered structure. Although a volcano plot was not directly constructed, the PDOS and adsorption energy analyses demonstrated a strong correlation between the d-band center and catalytic activity, consistent with the expectation of the Sabatier principle that moderate adsorption was optimal. The downshift of the d-band effectively modulated the adsorption of reaction intermediates: the CO* adsorption energy (Figure 11c,d) decreased from −2.78 eV (D) to −1.55 eV (O), weakening the binding of toxic intermediates and alleviating Pt poisoning; the OH adsorption energy (Figure 11e,f) decreased from −2.91 eV (D) to −3.42 eV (O), and the stronger OH* adsorption enhanced the bifunctional mechanism, accelerating the oxidative removal of CO and other species. Furthermore, the energy barrier for the rate-determining step of the EOR (the C–C bond cleavage) was calculated (Figure 11g,h). The barrier on O–Pt_3_Fe was only 0.67 eV, much lower than the 0.83 eV on D-Pt_3_Fe, indicating that the ordered structure facilitated the complete oxidation of ethanol. The above theoretical calculations were in good agreement with experimental observations: the MA of O-Pt_3_Fe/rGO reached 5.66 A/mg, which was 7.1 times that of commercial Pt/C, and it retained 80.7% of its activity after 500 cycles. The study revealed, at the atomic level, that ordered intermetallic compounds synergistically optimized the electronic structure through strain and ligand effects, while simultaneously weakening CO* adsorption, enhancing OH* supply, and lowering the C–C cleavage energy barrier, thereby providing a solid theoretical foundation for the rational design of high-performance platinum-based electrocatalysts.

**Figure 12:**
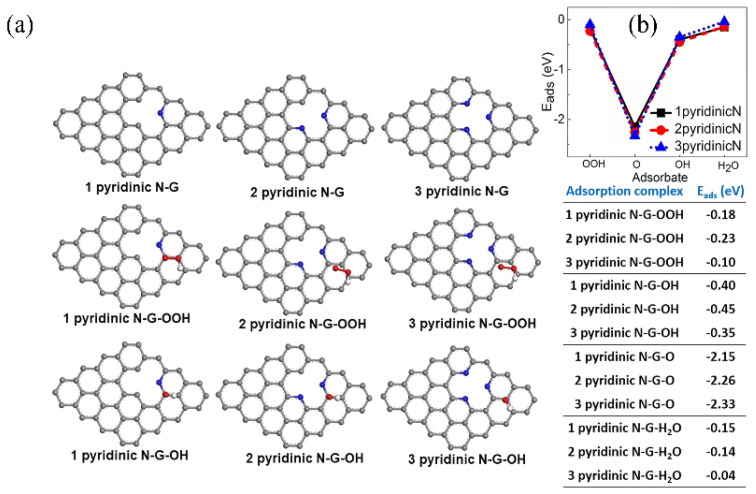
The ORR (**a**) and OER (**b**) GFE diagrams of elementary reactions for NC, CoS_2_ and CoS_2_/NC [123]. Copyright 2023, Royal Society of Chemistry.

Tu et al. [123] synthesized a CoS_2_/NC bifunctional electrocatalyst via a low-temperature molten salt template method. The DFT calculations showed that for the ORR, the rate-determining step of NC was the formation of *OOH, that of CoS_2_ was the reduction of *O to *OOH, while that of CoS_2_/NC was the desorption of *OH, exhibiting the lowest overpotential (Figure 12). For the OER, the rate-determining step of CoS_2_/NC was the conversion of *O to *OH, with an overpotential of only 0.651 V, which was much lower than those of NC and CoS_2_. Moreover, CoS_2_/NC possessed a stronger oxygen adsorption ability and a lower work function, which facilitated oxygen activation and electron conduction. The DFT calculations confirmed the synergistic effect between CoS_2_ and NC from the perspectives of thermodynamics and electronic structure.

Although this review focuses on platinum-based catalysts, significant progress has also been made in recent years in the field of fuel cells using non-noble metal (transition metal sulfides) and metal-free (heteroatom-doped carbon materials) catalysts. These studies provide important insights for understanding catalytic mechanisms and designing efficient electrocatalysts, while also revealing potential directions for further optimizing platinum-based catalysts.

Currently, alternative materials represented by palladium-based catalysts, non-noble metal catalysts, and heteroatom-doped carbon materials have shown promising prospects in the field of low-cost fuel cells. Although these materials differ from platinum-based catalyst systems, they provide important inspiration for the design of next-generation fuel cell catalysts. For next-generation electrocatalysts, the development approach should not be limited to simple elemental substitution, but rather shift toward precise atomic and nanostructure regulation, optimization of the electronic structure of active sites, and rational design of three-phase interfaces with high stability and poison tolerance. Green synthesis methods, with their advantages of low energy consumption, high controllability, and non-polluting nature, provide an ideal platform for achieving the above goals. In the future, they will continue to drive innovation in novel catalyst systems that combine high activity, high stability, low cost, and sustainability, ultimately realizing the synergistic unification of performance and greenness.

## 3. Green Synthesis of Platinum-Based Catalysts: Structure–Performance Relationship and Electrochemical Kinetic Behavior

The catalytic performance of fuel cell catalysts is determined by their microstructure. The relationship between structure and performance depends on electrochemical kinetics and the adsorption-conversion mechanism of intermediates. Green synthesis strategies exhibit distinct merits including mild reaction conditions, free of surfactant contamination, and precise control over nucleation and crystal growth. These strategies can effectively regulate the size, morphology, and surface state of platinum-based catalysts, and further optimize active-site exposure, charge transfer efficiency, and structural stability. Accordingly, the intrinsic relationship among synthesis method, microstructure, and catalytic performance can be well established. In addition, external factors such as operating temperature and electrolyte environment also significantly influence catalytic reactions.

This chapter systematically discusses the structure-activity relationship and electrochemical kinetic behaviors of green-synthesized platinum-based electrocatalysts. Firstly, the kinetic regulation of particle size, morphology, and surface state on reactions such as ORR and MOR is analyzed, and the deficiencies of current studies in particle size distribution, Tafel slope comparison, in situ stability of high-energy surfaces, and quantitative correlation of surface cleanliness are identified. Secondly, the influence of temperature from cold start to high-temperature ranges on kinetics and stability is explored, clarifying the lack of systematic investigation over a wide temperature range. Finally, the differences in kinetics and reaction pathways between acidic and alkaline electrolytes are compared, identifying the insufficient analysis of kinetic origins in acid-base comparative studies. On this basis, the key scientific issues of green synthesis in kinetic quantitative research, in situ mechanism characterization, wide-temperature-domain and multi-environment adaptability are summarized, and the design principles of high-efficiency, stable, and green platinum-based catalysts for practical working conditions are proposed.

### 3.1. Regulation Mechanism of Particle Size on Catalyst Structure and Performance

Catalyst size is a key structural parameter for regulating fuel cell performance. It is directly related to the ECSA, the coordination state of surface atoms, and the electronic structure, thereby significantly influencing the MA, long-term stability, and precious metal utilization of core reactions such as ORR and MOR. The size effect primarily operates through geometric and electronic dual mechanisms: at the geometric level, reducing particle size increases the proportion of low-coordination atoms and modulates the exposure of active crystal facets, thereby enhancing the density of active sites; at the electronic level, the change in particle size induces the quantum confinement effect, which modifies the band structure and surface adsorption behavior of the catalyst, optimizes the adsorption strength of reaction intermediates, and reduces the reaction energy barrier [125].

However, a smaller particle size is not always better. When particles become too small (typically less than 2 nm), the surface energy increases significantly, which exacerbates nanoparticle agglomeration, Ostwald ripening, or dissolution under harsh electrochemical conditions, thereby severely compromising long-term stability [126]. Conversely, excessively large particles lead to a marked decrease in ECSA and noble metal MA [127]. Therefore, an optimal size distribution must strike a balance between high intrinsic activity and excellent durability, which is highly dependent on the precise control of nucleation and growth kinetics afforded by the preparation method. Green synthesis strategies, with their mild and controllable nature, offer an effective pathway to achieve this balance.

**Figure 13:**
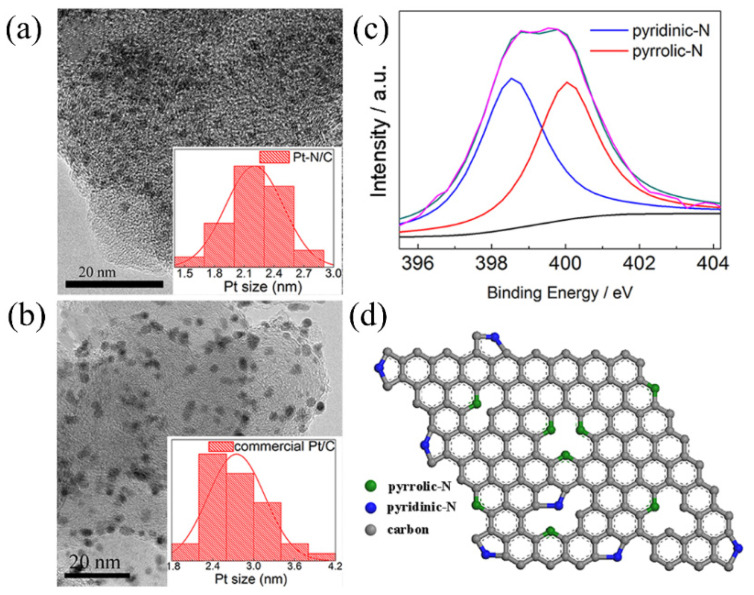
TEM images of (**a**) Pt-AL/C and (**b**) commercial Pt/C, (**c**) XPS spectrum of N 1 s in Pt-AL/C, and (**d**) illustration of N dopants [129]. Copyright 2018, American Chemical Society.

**Figure 14:**
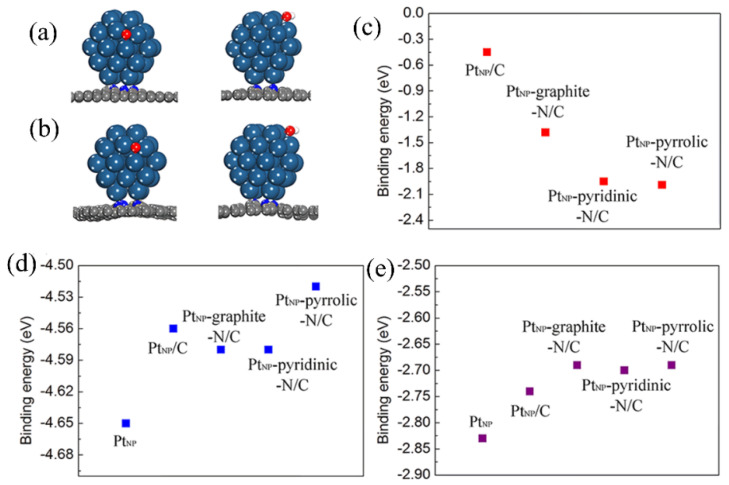
DFT-optimized geometries of *O and *OH on (**a**) Pt_NP_-pyridinic-N/C and (**b**) Pt_NP_-pyrrolic-N/C, (**c**) binding energies (in eV) of Pt_NP_ (Pt = 38) on different supports, and (**d**) OBE and (**e**) OHBE of different models [129]. Copyright 2018, American Chemical Society.

For pure platinum catalysts, their activity and stability are highly dependent on particle size and dispersion. Paperzh et al. [128] utilized UV radiation to introduce additional nucleation sites in situ within a carbon black suspension, which not only reduced the average particle size of the Pt nanoparticles but also optimized their size distribution and spatial dispersion. Using formic acid as a reducing agent, UV radiation increased the ECSA of the catalyst by up to 80%, with the optimal sample, F_UV/C_^80^, achieving an MA of 310 A/gPt for ORR at 0.9 V, significantly outperforming both the non-irradiated samples and commercial catalysts. This study demonstrated the potential of UV radiation as an efficient and clean physical modulation approach for simultaneously optimizing catalyst microstructure and enhancing intrinsic activity. In addition to physical energy modulation, green templates also represented an effective strategy for achieving ultrafine dimensions and high stability in pure platinum catalysts. Wang et al. [129] utilized L-phenylalanine (LPHE) as a green template and dispersant to fabricate Pt-LPHE films via a room-temperature electron reduction (RTER) method, where the Pt NPs were highly dispersed and predominantly exposed (111) facets. Loading this film onto a carbon support yielded the Pt-AL/C catalyst with an average particle size of 2.1 nm and enriched (111) facets, simultaneously achieving the highly dispersed loading of Pt NPs and nitrogen doping (Figure 13a–d). DFT calculations demonstrated that the nitrogen dopants in the support formed strong interactions with Pt, which not only stabilized the Pt NPs but also reduced the adsorption energies of surface *O/*OH intermediates, optimizing the reaction pathway (Figure 14a–e). Electrochemical test results showed that the MA of this catalyst for the ORR was 2.7 times that of commercial Pt/C, and the MA only decreased by 7% after 10,000 cycles, exhibiting significantly enhanced activity and stability. Through the synergistic effect of physical confinement and electronic modulation, this study provided a green and energy-neutral novel approach for preparing metal nanocatalysts with controllable structures and excellent performance.

In addition, alloying Pt with non-precious metals enables particle size reduction while synergistically modulating the electronic structure, further breaking through the performance limitations of pure platinum systems. Lobos et al. [130] employed a pulsed microwave-assisted reduction method to prepare a PtNi catalyst with an average particle size of 2.7 nm on phosphoric acid-activated cork kraft pulp-derived biocarbon (TBK), with a Ni content of approximately 30 at%, 61% of which formed an alloy. The high specific surface area and abundant oxygen-containing functional groups of the phosphoric acid-activated biocarbon enhanced the dispersion of PtNi. Combined with the small-size effect and the synergistic action of the electronic effect from alloyed Ni and the bifunctional mechanism from non-alloyed Ni, the catalyst achieved ethanol oxidation peak current densities of 476 A/g_Pt_ at 25 °C and 3574 A/g_Pt_ at 60 °C, which were 4.4 and 3.7 times higher than those of commercial Pt/C, respectively. Moreover, it exhibited excellent long-term stability, maintaining a steady current density more than 6.3 times that of commercial Pt/C after 4500 s of CA testing. Das et al. [131] employed a sequential supercritical carbon dioxide deposition technique to prepare uniformly dispersed PtNi, PtFe, and PtCu alloy catalysts supported on graphene, with particle sizes ranging from 3 to 5 nm. Among them, the PtNi/GNPs catalyst exhibited the best hydrogen oxidation reaction (HOR) and ORR activities, benefiting from the size effect, alloying effect, and a more favorable four-electron transfer pathway for the ORR. It also showed superior stability compared to the other alloy catalysts. In single-cell PEMFC tests, when PtNi/GNPs was used as the anode, it achieved a current density of 907.5 mA/cm^2^ at 0.6 V and a maximum power density of 0.54 W/cm^2^, outperforming commercial Pt/C and the other alloy catalysts.

### 3.2. Regulation Mechanism of Morphology on Catalyst Structure and Performance

The catalytic activity, selectivity, and stability of a catalyst are closely related to its microscopic morphology. The morphology directly affects the number, distribution, and atomic coordination environment of surface-active sites, with different morphologies typically corresponding to distinct surface atomic arrangements and coordination structures [132]. Through systematic morphology engineering, both the geometric structure and electronic properties of the material can be optimized, allowing for the selective exposure of highly active crystal facets and defect sites. Furthermore, the quantum confinement effects and changes in the surface coordination environment induced by morphology can further modulate the d-band center and spatial charge distribution. This enables regulation of the orbital overlap between reactant molecules and the catalyst, as well as the interfacial charge transfer kinetics, ultimately determining the pathway and efficiency of the catalytic reaction [133]. Therefore, achieving precise morphological control is key to enhancing the overall performance of platinum-based catalysts. Green synthesis methods provide an environmentally friendly route to achieve morphology-controlled synthesis, enabling fine regulation of crystal growth kinetics under mild conditions. In this way, high-performance catalysts with ideal surface structures can be constructed, promoting the synergistic development of fuel cell catalysts toward both high performance and sustainability.

Early research focused on using biomacromolecules or organic ligands as templates or directing agents to achieve the ultimate dispersion of nanoparticles and the exposure of specific crystal facets. For example, the Song group [134] utilized the spatial confinement effect of bovine serum albumin to uniformly deposit Pt nanoparticles onto the surface of porous, microtubular Zn_3_(PO_4_)_2_@BSA. The resulting Pt nanoparticles had an average particle size of only 1.32 nm and ECSA as high as 149.11 m^2^/g. Their MA for MOR reached 725.56 mA/mg_Pt_, which was 4.4 times higher than that of a Pt catalyst supported on multi-walled carbon nanotubes (PtNP@MWCNT). Additionally, this catalyst exhibited a low onset potential for methanol oxidation of 0.23 V, along with excellent CO tolerance and long-term stability. It maintained a relatively stable current density even after a 3000-s CA test.

The Shi group [135], using L-glutamic acid as a shape-directing agent, successfully synthesized a sheet-like PtPd alloy network enriched with highly active (111) crystal facets. This catalyst achieved the SA for methanol oxidation that was 38 times higher than that of commercial Pt black. Its half-wave potential for the ORR was shifted positively by 50 mV compared to Pt black. After 1000 cycles, its half-wave potential shifted negatively by only 3 mV, demonstrating excellent stability. These works confirmed that ultrafine dispersion and crystal facet engineering could significantly enhance catalytic performance. However, these methods suffered from long synthesis times, complex processes, and strong reliance on specific raw materials, which limited their large-scale application.

**Figure 15:**
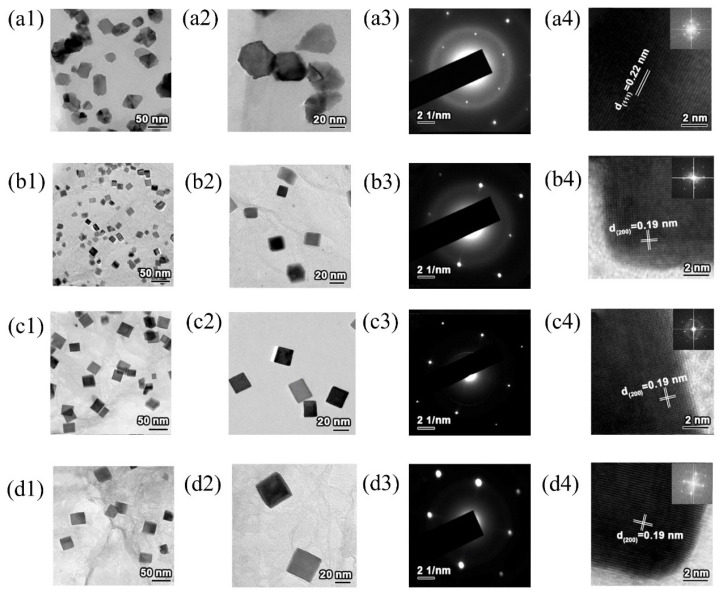
TEM images of the as-prepared PtPd NSs/RGO (**a1**,**a2**), PtPd NCs/RGO (**b1**,**b2**), PtCu NHCs/RGO (**c1**,**c2**), and PtPdCuNCs/RGO (**d1**,**d2**). SAED patterns of the single PtPd NS/RGO (**a3**), PtPd NC/RGO (**b3**), PtCu NHC/RGO (**c3**), and PtPdCu NC/RGO (**d3**). HRTEM images of PtPd NSs/RGO (**a4**), PtPd NCs/RGO (**b4**), PtCu NHCs/RGO (**c4**), and PtPdCu NCs/RGO (**d4**); insets in the HRTEM images are the corresponding FFT images, respectively [139]. Copyright 2017, American Chemical Society.

**Figure 16:**
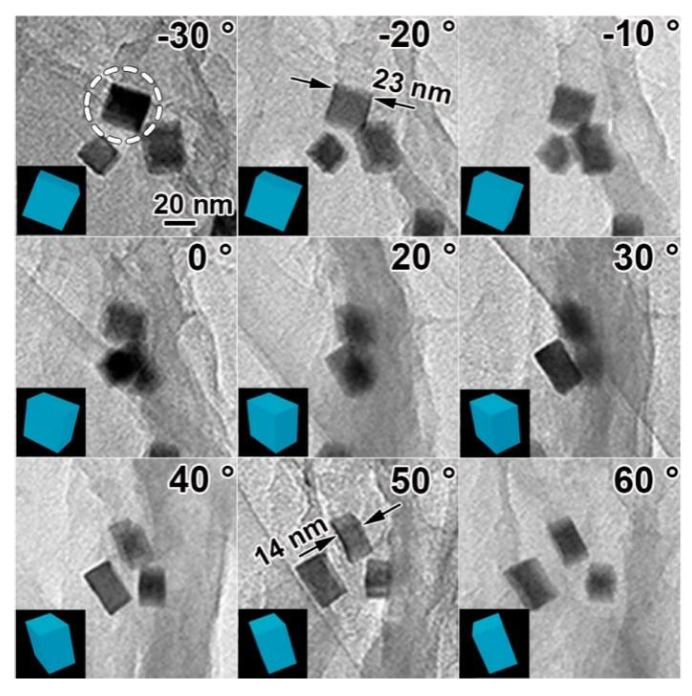
A set of TEM images of PtCu NHCs/RGO obtained at different tilting angles with X as the axis of rotation from −30 to 60°; the scale bar applies to all images, and the insets in the lower left corner show the corresponding models of the PtCu NHC in the white circle region [139]. Copyright 2017, American Chemical Society.

To improve preparation efficiency, research shifted toward cleaner in situ synthesis strategies, with a focus on constructing one-dimensional, two-dimensional, and three-dimensional low-dimensional structures. The Xie group [136] and the Xin group [137] both employed a formic acid reduction method at room temperature to grow Pt nanowires and ultrathin Pt layer in situ on a titanium substrate and on carbon paper, respectively, achieving excellent cell performance while significantly reducing the platinum loading. The Liu group [138] constructed dendritic Pd-Pt alloy catalysts directly on carbon paper via a surfactant-free electrochemical method. The dendritic structure is rich in unsaturated atoms and atomic defects, with exposed (111) active crystal planes. Its MA for formic acid oxidation reached 0.77 A/mg, which was 2.5 times that of commercial Pd/C, and its stability in the 3000-s CA test was 4 times that of commercial Pd/C. The in situ growth mode eliminated the need for binders and catalyst transfer. This not only significantly simplified the electrode preparation process and enhanced the bonding strength between the catalyst and the substrate but also maximized the utilization of active sites, effectively improving catalytic performance. Qiu et al. [139] employed an inorganic molten salt method in an organics- and surfactant-free system, using GO as a stabilizer and KI as a shape-inducing agent, to successfully achieve morphology-controlled synthesis of platinum-based catalysts. Figure 15 showed that they prepared a variety of morphologies, including PtPd NSs/RGO (nanosheets), PtPd NCs/RGO (core–shell nanocubes), PtCu NHCs/RGO (nano-hemicubes), and PtPdCu NCs/RGO (trimetallic nanocubes). Figure 16 further confirmed the three-dimensional shape of the PtCu NHCs/RGO nano-hemicubes through tilt-angle TEM. Among the as-obtained catalysts, the PtPd NCs/RGO nanocubes with a core–shell structure exhibited excellent catalytic activity and stability for methanol electrooxidation.

Ishak et al. [140] used sugarcane bagasse extract as a reducing and capping agent to achieve morphology-controlled synthesis of Pt nanoparticles. When the precursor concentration increased from 0.5 mM to 1.5 mM, the Pt NPs evolved from spherical (3.85–4.75 nm) into anisotropic structures such as cubes, triangles, and rhombuses (6.15 nm). Upon further increasing the concentration to 1.75 mM, the anisotropic structures became more pronounced, but severe particle agglomeration occurred, the particle size increased to about 13.47 nm, and the size distribution broadened significantly. Among these, Bio-Pt_1.5_, owing to its anisotropic structure and high proportion of metallic Pt, achieved the ECSA of 93.41 m^2^/g_Pt_ and a MA for MOR of 581.50 mA/mg_Pt_, which were 3.4 and 3.67 times those of commercial Pt black, respectively, while also exhibiting the best stability.

In recent years, research has further advanced toward the rational construction of multi-component alloys and heterogeneous interfaces, realizing functional zoning and synergistic enhancement at the atomic scale. The Kumar group [141] designed a layered heterogeneous structure with an inner Au layer and an outer Pt layer via sequential electrodeposition. In an alkaline system, the surface Pt layer was responsible for methanol adsorption and oxidation, while the underlying Au layer promoted the oxidative removal of CO intermediates through the electronic effect. This synergistically improved catalytic activity and anti-poisoning ability from the mechanism level, with overall performance comprehensively superior to that of commercial Pt/C catalysts. The methanol oxidation activity was increased by nearly 2 times, and the ECSA reached 204.15 m^2^/g_Pt_. This progress marked that the design of platinum-based catalysts has moved from single morphology regulation to a new stage of rational construction of multi-functional heterogeneous structures based on electrocatalytic reaction mechanisms.

### 3.3. Regulation Mechanism of Surface State on Catalyst Structure and Performance

Surface state is an atomic-level factor that determines the performance of platinum-based catalysts. Precise regulation of surface atomic arrangement, electronic structure, and chemical environment is the key to achieving high activity, high selectivity, and long durability. This can mainly be realized through three core strategies: defect engineering, surface cleanliness, and surface coating [142,143,144]. The mildness, controllability, and sustainability of green preparation technologies provide unique advantages for the precise construction of these surface states.

**Figure 17:**
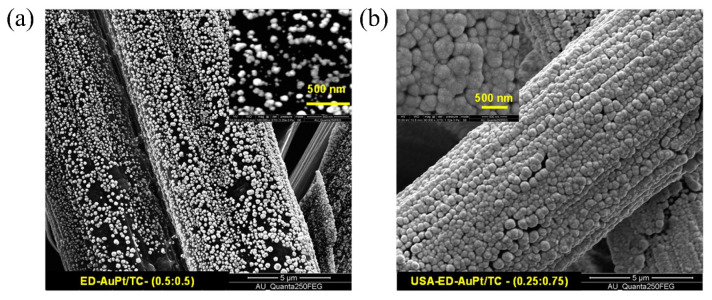
FE-SEM images of deposited AuPt nanostructured particles on TC substrate by (**a**) ED and (**b**) USA-ED methods. (Insets: higher magnification of corresponding images) [145]. Copyright 2014 American Chemical Society.

**Figure 18:**
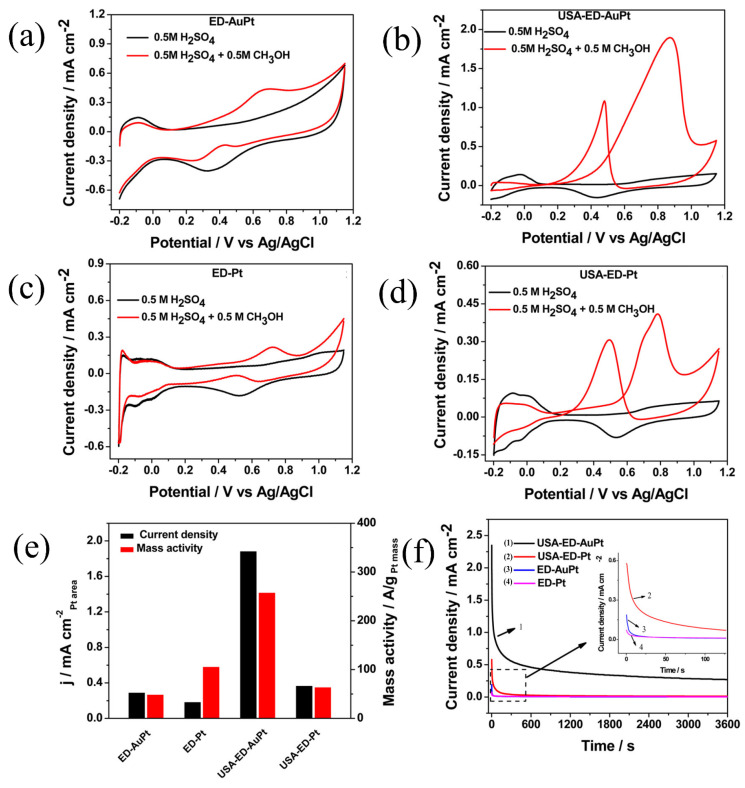
CVs of (**a**) ED-AuPt, (**b**) USA-ED-AuPt, (**c**) ED-Pt, and (**d**) USA-ED-Pt nanostructured surfaces on GCE substrate in presence/absence of 0.5 M methanol containing 0.5 M H_2_SO_4_ solution at the scan rate of 0.1 V/s. (**e**) Bar chart for comparison of specific activities and mass activities of deposited Pt and AuPt nanostructured surfaces at various conditions. (**f**) i−t curve response for USA-ED-AuPt, USA-ED-Pt, ED-AuPt, and ED-Pt in the mixture of 0.5 M H_2_SO_4_ + 0.5 M methanol at constant potential of 0.675 V [145]. Copyright 2014 American Chemical Society.

Defect engineering can introduce high-energy defects such as lattice vacancies, dislocations, or grain boundaries. It breaks the long-range order of surface atoms, creates a large number of coordination-unsaturated active sites, and optimizes reaction pathways. Green physical methods have prominent advantages in constructing clean defects. Boopathi et al. [145] utilized the ultrasonic cavitation effect to successfully prepare a densely packed, Pt-rich AuPt alloy nanostructure on a carbon substrate via ultrasonic-assisted electrodeposition (Figure 17). Compared with the loosely packed, homogeneous AuPt alloy (Au:Pt = 0.5:0.5) obtained by conventional electrodeposition, the ultrasonic-assisted electrodeposition induced Pt surface segregation (Au:Pt = 0.25:0.75), forming a cauliflower-like nanostructure. Benefiting from the ensemble effect of Pt surface segregation and the electronic synergy between Au and Pt, the resulting catalyst comprehensively outperformed commercial Pt/C in terms of the SA, MA, and long-term stability for methanol oxidation in acidic systems (Figure 18). The ultrasonic-assisted electrodeposition also likely introduced a high density of surface defects. Zheng et al. [146] prepared a PtCo/C-700 catalyst using wet ball milling combined with annealing at 700 °C under an H_2_/N_2_ atmosphere. EXAFS revealed that the catalyst possessed a high Pt–Co coordination number, short bond length, and strong compressive strain, along with abundant defects and low-coordination active sites at the edges and corners. DFT calculations confirmed that the synergistic effect of electronic interactions and compressive strain weakened the adsorption of phosphate species, thereby enhancing poisoning tolerance. Combined with the rapid dissociation of *OOH intermediates confirmed by in situ ATR-SEIRAS, the catalyst optimized the ORR pathway, resulting in a much lower overpotential than that of pure Pt. This synergy between surface electronic structure modulation and defect engineering significantly enhanced the oxygen reduction kinetics and phosphate tolerance. Consequently, PtCo/C-700 exhibited excellent electrocatalytic performance in high-temperature proton exchange membrane fuel cells. Its MA at 0.6 V was 2.2 times that of commercial Pt/C, and it showed only a 1.2% performance decay after a 168 h durability test.

Surface cleanliness directly determines the exposure degree of Pt active sites and is a core factor affecting electrocatalytic efficiency and noble metal utilization. Ishak et al. [147] achieved the green one-pot synthesis of Pt NPs, using extracts from agricultural wastes such as sugarcane bagasse, pineapple peel, and banana peel as both reducing agents and capping agents. TGA tests showed that surface cleanliness could be quantified by the weight loss of organic residues: Pt NPs synthesized with sugarcane bagasse extract had the least surface biomolecular residues, with a weight loss of only 8.64%, while the banana peel system had the most residues, with a weight loss of 22.85%. In the acidic methanol oxidation system, the residual surface biomolecules covered Pt active sites, leading to insufficient exposure of active sites. Thus, electrochemical tests demonstrated that the sugarcane bagasse system had an ECSA as high as 94.58 m^2^/g_Pt_ and a MA of 398.20 mA/mg_Pt_, whereas the ECSA of the banana peel system dropped sharply to 9.91 m^2^/g_Pt_. This study revealed that surface cleanliness was a key link connecting green preparation methods and catalytic performance.

Surface coating constructs a physical protection layer and an interfacial electronic modulation system. This strategy can inhibit the agglomeration and electrochemical dissolution of Pt particles, while also optimizing the surface electronic state through interfacial interactions. Zhang et al. [148] used renewable chitosan as a carbon and nitrogen source. After freeze-drying and carbonization, they introduced a PProDOT conductive polymer coating via in situ polymerization. This composite coating stabilized the Pt particles through π-π interactions, resulting in smaller Pt nanoparticles with more uniform dispersion. The nitrogen doping characteristics of the coating, along with the Schottky junction formed with Pt, further optimized the electronic structure of Pt and weakened CO adsorption strength. As a result, the catalyst achieved a MA for methanol oxidation that was 3.5 times higher than that of commercial Pt/C. It also retained 90% of its current density after 500 cycles, demonstrating significantly enhanced CO tolerance and cycling stability.

### 3.4. Electrochemical Kinetic Behaviors and Influencing Factors of Green-Synthesized Platinum-Based Catalysts

#### 3.4.1. Regulation of Catalyst Structure on Electrochemical Kinetic Behaviors

Although the relationship between particle size and catalytic activity has been widely recognized, most studies on green synthesis only report the average particle size rather than the full particle size distribution. From the perspective of electrochemical kinetics, a broad particle size distribution accelerates Ostwald ripening during long-term operation, thereby speeding up catalyst deactivation [149,150]. Systematic comparisons of Tafel slopes among catalysts with different size distributions remain scarce. Similarly, high-index facets and abundant step, edge, and defect sites are often used to explain the enhanced activity of morphologies such as nanoflowers, nanowires, dendritic structures, and nanocages [151,152]. However, the current primary goal of green synthesis methods remains the controlled synthesis of specific morphologies and the validation of their activity, rather than in-depth mechanistic and quantitative kinetic studies. Yajima et al. successfully distinguished CO adsorption on Pt sites from water activation on Ru sites during methanol oxidation using in situ ATR-FTIR, and detected the COO^−^ intermediate. This revealed intermediate information and site partitioning that cannot be obtained by electrochemical measurements alone, demonstrating that in situ spectroscopy is an indispensable tool for elucidating reaction pathways and adsorption sites [153]. Although a high *I_f_*/*I_b_* ratio suggests a higher oxidation removal efficiency of CO intermediates, it cannot distinguish between changes in reaction pathways and increased OH^−^ adsorption sites, requiring further investigation combined with in situ spectroscopic techniques such as in situ FTIR or Raman. Moreover, the morphological stability of high-energy surfaces under realistic operating conditions lacks systematic in situ verification. Without such data, the claimed durability advantages of specific morphologies remain at a qualitative level. Surface cleanliness is a prominent advantage of green routes such as molten salt, supercritical fluid, and biosynthesis methods, which do not use strong surfactants or capping agents. From a kinetic perspective, a clean surface eliminates the blocking of active sites and mass transfer resistance caused by organic residues, thereby enhancing intrinsic activity and charge transfer efficiency [154]. However, such “naked” nanoparticles are generally more prone to agglomeration and dissolution. Therefore, it is necessary to systematically explore weakly adsorbed and easily removable green ligands that can stabilize particles without impairing kinetics, so as to achieve a balance between activity and stability. Electrochemical impedance spectroscopy (EIS) can decouple the charge transfer resistance (Rct) from ohmic resistance and mass transfer resistance, providing a direct kinetic fingerprint for identifying surface structural effects [155,156].

#### 3.4.2. Mechanism of the Influence of Temperature on Electrochemical Kinetics and Stability

The actual operating temperature of fuel cells varies depending on the type. Proton exchange membrane fuel cells (PEMFCs) typically operate at 60 to 80 °C, whereas high-temperature PEMFCs (HT-PEMFCs) operate at 120 to 200 °C [157]. In addition, cold-start conditions of vehicles require that the catalyst maintain sufficient activity at subzero temperatures [158]. Therefore, systematically investigating the electrochemical performance of platinum-based catalysts over a wide temperature range is of crucial importance for practical applications.

Xu et al. [28] evaluated the performance of the PtPd/GO catalyst toward alkaline methanol oxidation at a low temperature of 5 °C, and found that it retained approximately 70% of its room-temperature activity, while commercial Pt/C retained only 57%. It maintained 56.7% of its initial activity after a 10,000-s durability test, rendering it suitable for fuel cell applications under extreme low-temperature conditions. Notably, 5 °C remained above the freezing point, and genuine subzero cold-start conditions have not been investigated. Zha et al. [109] investigated the cell performance of p-PtNi/p-NCFs@CC as the cathode in alkaline direct methanol fuel cells (ADMFCs) at 25, 40, 60, and 80 °C. The peak power density increased from 24.8 mW/cm^2^ at 25 °C to 103.9 mW/cm^2^ at 80 °C, far exceeding that of commercial Pt/C (15.4–50.3 mW/cm^2^). Furthermore, Zheng et al. [146] prepared a PtCo/C-700 catalyst via mechanical ball milling, which achieved a peak power density of 0.401 W/cm^2^ in HT-PEMFCs at 160 °C, with only 1.2% performance degradation after 168 h of operation, demonstrating excellent high-temperature stability. Although the first two studies were conducted in alkaline systems, whereas PEMFCs typically operated in acidic environments, the regulatory effect of temperature on electrocatalytic reaction rates was universal.

The above studies have experimentally confirmed the significant influence of temperature on catalytic performance, and the underlying reasons can be understood from the principles of electrochemical kinetics. From the perspective of fundamental electrochemical kinetics, the effect of temperature on reaction rate primarily follows the Arrhenius law [159]. Increasing the temperature generally enhances the diffusion coefficient of reactants and the conductivity of the electrolyte, reduces the overpotential, increases the exchange current density, and may alter the adsorption equilibrium of reaction intermediates [160]. However, excessively high temperatures also accelerate catalyst degradation, leading to a decline in long-term stability [161]. Therefore, for each catalyst, there is typically an optimal operating temperature range, where a balance between activity and stability must be achieved.

Although the studies mentioned above provide data at a limited number of temperature points, overall, the vast majority of green synthesis research still uses room temperature (approximately 25 °C) or a single temperature as the main evaluation condition. These studies lack systematic investigation over a wide temperature range (especially subzero and actual operating temperatures). They also fail to perform Arrhenius analysis based on kinetic currents at different temperatures to extract the apparent activation energy (E_a_). Consequently, it is impossible to accurately determine whether green synthesis strategies truly lower the reaction energy barrier, rather than merely relying on activity comparisons at room temperature. Therefore, future research should systematically measure the polarization curves and Tafel slopes of green synthesis catalysts at a series of temperatures, including −20, 0, 25, 40, 60, 80, 120, 160, and 200 °C. The apparent activation energy should then be calculated using Arrhenius plots.

#### 3.4.3. Regulation Mechanism of Electrolyte Environment on Electrochemical Kinetics and Reaction Pathways

The electrocatalytic performance and electrochemical kinetics of platinum-based catalysts differ significantly in different electrolyte environments. These differences play a key regulatory role in reaction pathways, intermediate adsorption behavior, poisoning tolerance, and long-term stability. In acidic systems, the reactions are dominated by proton-coupled electron transfer. The MOR and EOR mainly follow the CO pathway, where strong adsorption of CO intermediates on the Pt surface poisons active sites, and poisoning tolerance typically relies on the bifunctional or electronic effects provided by alloy components [162]. In addition, anions such as sulfate may specifically adsorb on the Pt surface, further reducing the accessibility of active sites [163]. The ORR proceeds via a 4e^−^ pathway to produce water but exhibits slow kinetics; meanwhile, Pt is prone to dissolution and Ostwald ripening at high potentials [164]. In alkaline systems, OH^−^ directly participates in the catalytic process, allowing CO intermediates to be oxidatively removed at lower potentials. As a result, MOR/EOR often exhibit higher mass activities and more negative onset potentials, and the reaction pathway tends toward complete oxidation. However, for ORR, the strong adsorption of OH^−^ on the Pt surface occupies active sites, leading to slower kinetics (higher overpotential) than in acidic media, and such excessive adsorption may also hinder the access of other reactants [164,165].

For instance, Nie et al. [112] reported ordered O-Pt_3_Fe/rGO for ethanol oxidation in alkaline media, whose outstanding performance was attributed to OH^−^ participation and suppression of CO poisoning by the ordered structure, with a mass activity as high as 5.66 A/mg_Pt_, far superior to commercial Pt/C. The study also tested the catalyst under acidic conditions, achieving a mass activity of 1.39 A/mg_Pt_, which was significantly lower than that in alkaline media. This indicated that the catalyst was more suitable for alkaline environments. However, the authors did not perform an in-depth analysis of the kinetic origins of the performance differences between acidic and alkaline conditions. Zha et al. [109] tested a defective PtNi catalyst in 1 M KOH. It exhibited excellent MOR mass activity (1219 mA/mg_Pt_), attributed to the promotion of OH^−^ generation by Ni, which accelerated CO oxidation and enhanced poisoning tolerance. The catalyst also showed good ORR bifunctional activity (onset potential of 1.15 V, half-wave potential of 0.90 V), but no acidic reference data were provided. Some studies were conducted only in a single system, while the few that performed acidic-alkaline comparisons failed systematically reveal the kinetic origins (Tafel slopes, apparent activation energies, reaction orders).

Future research should systematically investigate the same green synthesis catalyst under identical testing conditions in both acidic and alkaline media, including cyclic voltammetry, linear sweep voltammetry, chronoamperometry, and CO stripping experiments. Tafel slopes should be determined to assess whether the rate-determining step is affected by pH. In situ infrared spectroscopy (ATR-FTIR) or Raman spectroscopy should also be employed to monitor differences in reaction intermediates.

## 4. Conclusions and Outlook

This review summarized the recent advances in the green synthesis of platinum-based catalysts for fuel cells, including green solvent systems, biosynthesis, renewable resource templates, and energy-saving methods. It was shown that green synthesis enabled precise regulation of the particle size, morphology, and surface state of platinum-based catalysts, thereby significantly enhancing their electrocatalytic activity, stability, and poison tolerance, and clearly established a structure–activity relationship among the green synthesis route, microstructural characteristics, and electrochemical performance.

However, the translation of this field from laboratory research to industrial application still faces multiple challenges. First, the long-term durability of catalysts prepared by green methods under harsh operating conditions, along with their full lifecycle cost advantages, has not yet been fully validated. Second, key bottlenecks for large-scale production remain, including compositional fluctuations in biological reduction methods, field uniformity control in microwave and plasma processes, and the engineering scale-up of molten salt and supercritical fluid technologies. Third, the microscopic mechanisms of most green synthesis processes remain unclear, due to a lack of systematic support from in situ characterization techniques and theoretical calculations. Fourth, the absence of a full lifecycle assessment framework makes it difficult to objectively evaluate the true environmental benefits of these methods.

In the future, the development of this field should take the low-carbon full lifecycle as the core driving force, pursuing synergistic breakthroughs across four dimensions. First, it is urgently necessary to establish a full lifecycle assessment framework to objectively evaluate the true environmental benefits of green synthesis methods, and to systematically validate the durability and cost advantages of catalysts under long-term harsh operating conditions. Second, DFT calculations, molecular dynamics simulations, electrochemical kinetics and in situ characterization techniques should be employed to deeply reveal the structure-activity relationship and the microscopic mechanisms of the synthesis process. Thirdly, continuous production equipment should be developed to improve the batch-to-batch stability of large-scale preparation. Fourthly, the green concept should be extended to low-Pt and Pt-free systems, optimizing the compatibility between catalysts and membrane electrode assemblies to achieve tailored adaptation for different types of fuel cells. Meanwhile, multi-strategy integrated innovation should be embedded across all the above dimensions to overcome the limitations of single approaches and achieve synergistic enhancement.

In conclusion, with breakthroughs in the key technologies outlined above, green synthesis methods are expected to provide the fuel cell industry with a catalyst manufacturing solution that combines high performance, low cost, and sustainability. This will accelerate the commercialization of fuel cells and contribute to the establishment of a clean energy system.

## Figures and Tables

**Table 1:** Performance Comparison of Various Fuel Cell Catalysts.

Serial Number	Material	Method	Pt Mass Loading (wt%)	ECSA (m^2^/g)	MA (mA/mg)	E_1/2_ (V)	Electrolyte	Ref.
1	Commercial Pt/C	-	20	65.7	258.5	-	0.5 M H_2_SO_4_	[116]
2	Pt concave NPs	Molten salt	20	5.48	502	-	0.1 M HClO_4_ + 0.5 M HCOOH	[43]
3	Pt-CQD300	Phytosynthesis	82.5	98.89	688.25	-	0.5 M H_2_SO_4_ + 1.0 M CH_3_OH	[62]
4	PtRu/C-JH-1000-50	Rapid Joule Heating	6.32	239	705.9	-	0.1 M HClO_4_ + 1.0 M CH_3_OH	[114]
5	M1V5 (Pt/Vulcan)	Microwave	2.6	98	422	0.897	0.1 M KOH	[85]
6	PtFe/B-FeNC	Mechanochemical	7.8	84.9	2570	0.95	0.1 M HClO_4_	[117]
7	PtCo_3__HTS	Rapid Joule Heating	20	-	647	0.897	0.1 M KOH	[113]
8	L1_0_-PtCo/HMC	high-temperature pyrolysis	-	107.8	3980	0.957	0.1 M HClO_4_ + 1 M CH_3_OH	[118]
9	PtNi NWs	Solvothermal	38	29.31	1737	-	1.0 M KOH + 1.0 M CH_3_OH	[119]
10	Pt_1_Ru_3_@MCHS	Vacuum impregnation	0.52	149	5860	-	1.0 M KOH + 1.0 M CH_3_OH	[120]
11	Pd/NPC	Biomass-derived carbon supports	0	128.1	2818	-	0.5 M NaOH + 1 M CH_3_OH	[121]
12	DPARH	Microbial synthesis	0	55.66	2040	-	0.5 M H_2_SO_4_ + 0.5 M HCOOH	[122]
13	CoS_2_/NC	Molten salt	0	-	-	0.854 V	0.1 M KOH	[123]
14	NCS-rGO	Biomass-derived carbon	0	-	-	0.86 V	0.1 M KOH	[124]

Annotation: DPARH is 3D porous heteroatom-doped bio-PdAu/reduced GO (rGO) hybrid. No. 2–7: Green-prepared Pt-based fuel cell catalysts. No. 8–10: Non-green-prepared Pt-based fuel cell catalysts. No. 11–12: Green-prepared Pd-based fuel cell catalysts. No. 13–14: Green-prepared other fuel cell catalysts. FAOR is formic acid oxidation reaction.

## Data Availability

The data presented in this study are available upon request from the corresponding author.
